# Spatial transcriptome mapping identifies *Ppara-Anxa2* cross-talk in microplastic-induced hepatotoxicity

**DOI:** 10.1126/sciadv.aec8681

**Published:** 2026-06-17

**Authors:** Woncheol Jung, Hassan Abushukair, Nikhil Y. Patil, Felix Ampadu, Maryam Firouzi, Iulia Rus, Jinhyuk Choi, Sree Deepthi Muthukrishnan, Surendra Shukla, Stefano Tarantini, Anna Csiszar, Kamiya Mehla, Dongin Kim, Je-Hyun Yoon, Dowoon Kim, Juyang Kim, Jaehak Jung, Oxana Klementieva, Yatrik M. Shah, Eiji Yoshihara, Pankaj K. Singh, Aditya D. Joshi, Tae Gyu Oh

**Affiliations:** ^1^ Department of Oncology Science, College of Medicine, University of Oklahoma Health Campus, Oklahoma City, OK, USA.; ^2^ Department of Pharmaceutical Sciences, University of Oklahoma Health Campus, Oklahoma City, OK, USA.; ^3^ Department of Veterinary Physiology and Pharmacology, Texas A&M University, College Station, TX, USA.; ^4^ The Lundquist Institute for Biomedical Innovation at Harbor-UCLA Medical Center, Torrance, CA, USA.; ^5^ OU Health Stephenson Cancer Center, University of Oklahoma Health Campus, Oklahoma City, OK, USA.; ^6^ Oklahoma Center for Geroscience and Healthy Brain Aging, University of Oklahoma Health Campus, Oklahoma City, OK, USA.; ^7^ Department of Pathology, College of Medicine, University of Oklahoma Health Campus, Oklahoma City, OK, USA.; ^8^ Korea Institute of Analytical Science and Technology, Seoul, Republic of Korea.; ^9^ Medical Microspectroscopy Lab, Department of Experimental Medical Science, Faculty of Medicine, Lund University, Lund, Sweden.; ^10^ Department of Molecular and Integrative Physiology and Internal Medicine, Division of Gastroenterology, University of Michigan, Ann Arbor, MI, USA.; ^11^ David Geffen School of Medicine at University of California, Los Angeles, CA, USA.

## Abstract

Microplastics (MPs) are increasingly recognized as an urgent global health concern, with mounting evidence linking their bioaccumulation to chronic disease risks. The liver, as a central organ for metabolic homeostasis and detoxification, is particularly sensitive to sustained MP exposure. However, the mechanisms underlying hepatic responses to polyethylene (PE), the most prevalent yet relatively understudied plastic type, remain poorly defined. By performing combined bulk and spatial transcriptomic analyses, we observed that PE resulted in hepatic dysfunction in mice fed on standard or metabolic dysfunction-associated steatohepatitis (MASH)–inducing diets, driving both globally and spatially distinct transcriptional alterations, including a nuclear receptor (NR), *Ppara*. Spatial transcriptomics uncovered the cell type heterogeneity and gene expression patterns masked in bulk analyses. Spatial clustering uncovered inflammatory hotspots characterized by reduced cellular diversity and distinct PE-responsive gene signatures. Among these, NR signaling emerged as a key regulatory axis, with Ppara identified as a modulator. We further demonstrated that Ppara regulates *Anxa2*, a gene involved in damage response and tissue recovery, through enhancer and promoter binding. These findings offer insights into how PE disrupts hepatic homeostasis and underscores the value of spatial transcriptomics in elucidating environmental impacts on tissue organization and gene expression regulation. This study provides a molecular framework for understanding MP-associated disruption of liver pathophysiology and highlights potential targets for therapeutic intervention.

## Teaser

Polyethylene microplastic drives liver dysfunction and controls *Ppara*-*Anxa2* gene expression.

## INTRODUCTION

Over the past few decades, plastics have seamlessly integrated into nearly every facet of human life (*1*–*3*). Despite the substantial environmental cost of this convenience, global plastic production is projected to rise as demand surges across industries, with an additional 12.5 billion tons expected by 2050 (*2*, *3*). Plastic is designed and used for its durability and resistance to natural degradation. However, over time, it breaks down into microplastics (MPs) with a particle size of 1 to 5000 μm due to exposure to sunlight, heat, and mechanical forces (*4*, *5*). These MPs have spread throughout global ecosystems, being found in oceans, freshwater bodies, agricultural soils, and even the air we breathe (*6*–*10*).

Given the ubiquity of MP in the environment, humans are widely exposed to environmental MP through multiple routes, inhalation, ingestion, and dermal contact (*1*, *11*–*13*). Numerous studies have confirmed the presence of various MP types in a range of human specimens (*14*–*22*). For example, different types of plastics, with particle sizes of 700 nm or larger, including polystyrene (PS), polyethylene (PE), polypropylene (PP), polymethyl methacrylate, and polyethylene terephthalate (PET), were observed in whole blood samples collected from 22 healthy donors (*23*). Similarly, previous studies have also reported that MP is detected in the testes and the placenta (*24*, *25*). Although there is ongoing debate about how much plastic the average person consumes (*26*–*28*), it is widely acknowledged that humans inevitably consume MPs, leading to continuous exposure (*29*, *30*). For this reason, there are growing concerns about the potential impact of internal exposure to MPs on human health. Among the many organs affected by MP accumulation, particular attention has been given to the liver. Metabolic dysfunction-associated steatohepatitis (MASH) is a major liver disease induced by excess fatty acid contents associated inflammation in the liver (*31*, *32*). In recent years, several studies using multiple experimental models revealed that MP causes inflammation and stimulates innate immune responses, leading to dysfunctional liver lipid metabolism and contributing to MASH development (*33*–*41*).

In this context, nuclear receptors (NRs), a superfamily of ligand-dependent transcription factors, play a pivotal role in regulating hepatic metabolism, inflammation, and detoxification. Disruption of NR signaling has been linked to MASH. Among these receptors, peroxisome proliferator–activated receptor α (PPARα) is a central regulator of fatty acid oxidation and inflammatory response in the liver. Therapeutically, PPARα activation has been shown to alleviate hepatic steatosis and inflammation, highlighting its promise as a target for MASH treatment (*42*–*44*). Ongoing clinical studies investigating PPARα agonists underscore the need to understand how environmental factors like MPs (e.g., PE) may disrupt NR signaling and progression of liver disease.

Several studies have explored gene expression profiles in MP-treated livers, primarily using bulk RNA sequencing (RNA-seq) profiling, while data from single-cell resolution remain limited. Given the cellular and spatial heterogeneity of liver anatomy, it is increasingly important to resolve gene expression not only at the single-cell level but also within the spatial context of hepatic zonation. To address this, we leveraged spatially resolved single-cell gene expression profiling to define the impact of MPs within the liver and to improve our understanding of the correlation between MP exposure and MASH development, aiming to guide potential preventative strategies.

To bridge current knowledge gaps, we used PE, which is the most abundant MP detected in humans (*45*). We conducted spatial transcriptome profiling using the 10X Genomics’ Xenium platform to investigate the effects of PE on liver gene expression at the single-cell level, along with the precise contextual information relevant to liver architecture. Through this comprehensive approach, we defined spatially resolved alterations in hepatic cellular composition and microenvironmental transcriptional states induced by PE, revealing inflammation-associated niches and NR-driven gene programs. Notably, we found that PE exposure modulates NR signaling pathways and identified Ppara as a key regulator that modulates Anxa2, a gene implicated in damage response and tissue recovery. These findings aim to improve our understanding of systemic effects of PE on the overall liver function and, more specifically, its effect on NR expression and regulation in relation to liver disease progression.

## RESULTS

### In vivo exposure to PE MP induces hepatotoxicity in male mice

**Fig. 1.**
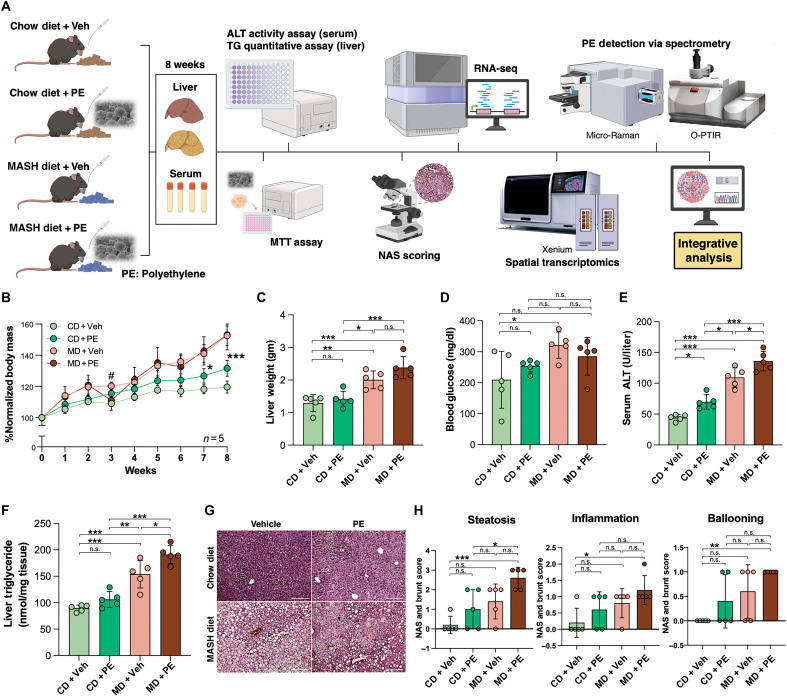
Molecular and histopathological effects of PE MPs in mouse liver with or without a high-fat diet. (**A**) A schematic overview of the experimental design including in vivo with RNA-seq and spatial transcriptome profiling, micro-Raman, and Optical Photothermal Infrared (O-PTIR). Created in BioRender O. (2026) https://BioRender.com/lalw3vm (**B**) Alteration of normalized body mass across treatment groups (*n* = 5 within each of the four groups). (**C**) Bar plot showing the percentage of liver weight relative to total body weight across treatment groups. Individual dots represent biological replicates. (**D**) Bar plot comparing blood glucose levels (milligrams per deciliter) among the different treatment groups. (**E**) Bar plot illustrating serum ALT activity (units per liter), a marker of liver injury, across groups. (**F**) Bar plot depicting liver TG content normalized to tissue weight (nanomoles per milligram) in each treatment group. (**G**) Representative hematoxylin and eosin (H&E)–stained liver sections displaying histopathological features from mice treated with CD + Veh, CD + PE, MD + Veh, and MD + PE groups, shown at ×40 magnification. The scale bar indicates a length of 180 μm. (**H**) Bar plots presenting steatosis, lobular inflammation, and hepatocellular ballooning scores in liver sections from mice across four groups as CD + Veh, CD + PE, MD + Veh, and MD + PE. Data are presented as mean ± SD. Statistical significance was determined using ordinary one-way analysis of variance (ANOVA) with Tukey’s multiple comparisons test. ns, not significant. *P < 0.05, **P < 0.01, and ***P < 0.001. Veh, vehicle; NAS, nonalcoholic fatty liver disease activity score.

To identify hepatotoxic MP types, we initially tested various types of MPs including PE, PS, PET, PP, polyvinylidene chloride (PVDC), polyamide-6 (PA-6), and polyvinyl chloride (PVC) in primary hepatocytes after 24 hours of exposure and found that PE resulted in the lowest cell viability (fig. S1A), with detailed experimental conditions and MP specifications described in Materials and Methods. To investigate the effect of PE on hepatotoxicity and metabolic dysfunction in vivo, wild-type (WT) mice fed either a control/CD (CD) or high-fat, high-fructose, high-cholesterol metabolic dysfunction-associated steatohepatitis (MASH) diet (MD) were subjected to oral PE administration (Fig. 1A). Mice fed a CD and gavaged with PE showed a gradual increase in body weight compared to the vehicle (Veh)-treated group, with a significant difference observed at the 7-week time point following daily oral PE gavage (2 mg/day), which was sustained throughout the experiment (Fig. 1B). Mice fed with MD showed a significant increase in body and liver weight compared to those fed the CD; however, treatment with PE did not impart any further increase in body mass in the MD-fed mice (Fig. 1B). Notably, administration of PE did not lead to an increase in liver weight in either CD or MD groups, although liver weight differed between CD and MD conditions (Fig. 1C and fig. S1B). The marked changes in body weight between the experimental groups were independent of glucose level (Fig. 1D). To confirm the presence of hepatic injury by PE, both alanine aminotransferase (ALT) and triglyceride (TG) levels were measured from the serum and liver, respectively. PE administration increased hepatic injury as measured by serum ALT levels in both CD- and MD-fed groups (Fig. 1E). Moreover, liver tissue from the MD + PE group showed elevated TG levels compared to the MD group (Fig. 1F). Histopathological assessment using the MASLD activity score was performed to assess the disease severity. This analysis demonstrated increased hepatic steatosis in PE-treated groups (Fig. 1G). Furthermore, an increasing trend in hepatocyte ballooning and inflammation was also observed in PE-treated groups compared to the CD-only cohort (Fig. 1H). Collectively, these results indicate that administration of PE for 8 weeks results in hepatotoxicity and exacerbates diet-induced steatohepatitis in male mice.

### Lipid metabolism–associated gene expression is significantly dysregulated in mouse liver following MD and PE exposure

**Fig. 2.**
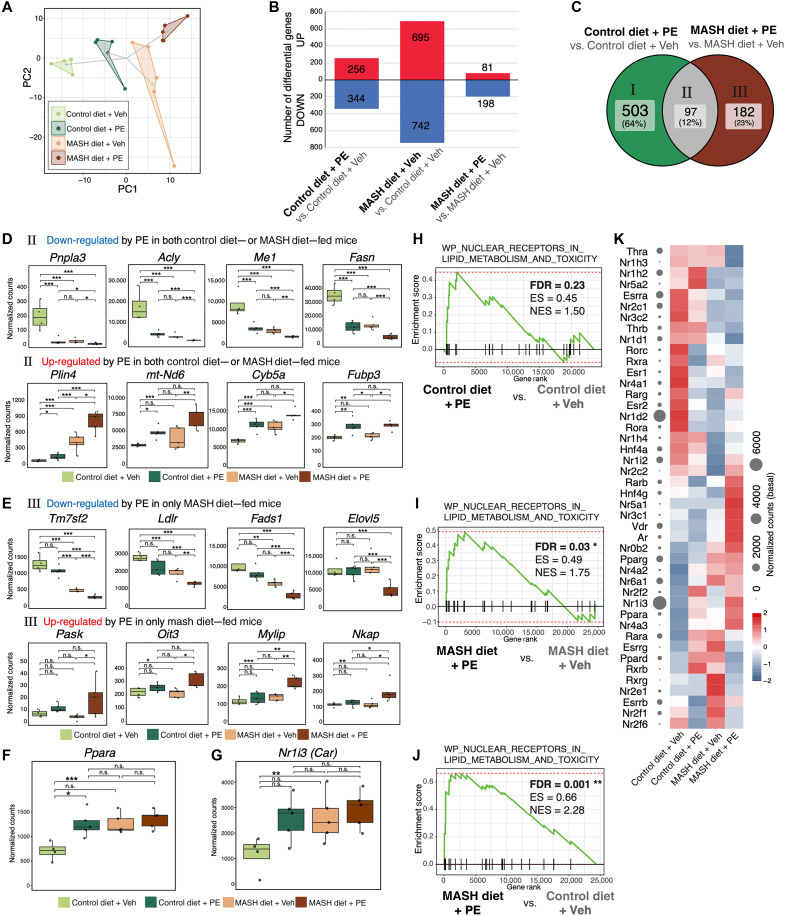
RNA-seq reveals that PE MP disrupts lipid metabolism and modulates hepatic toxicity pathways. (**A**) Bulk RNA-seq data show distinct transcriptional clustering by treatment group. (**B**) A pairwise identification of DEGs (|log_2_ FC| ≥ 0.38 and FDR < 0.05); up-regulated (red) and down-regulated (blue). (**C**) Venn diagram illustrating distinct and overlapping DEGs under CD (i.e., CD + PE versus CD + Veh) and MD (i.e., MD + PE versus MD + Veh) conditions, highlighting shared and diet-specific transcriptomic responses to PE treatment. (**D** and **E**) Normalized counts of DEGs responsive to PE treatment in both CD and MD conditions (D) or specifically in MD-fed mice (E). Boxplots (ggplot2) represent median, interquartile range (IQR), and whiskers (1.5× IQR). Pairwise differential expression analyses were performed across all condition comparisons, and results are reported as false discovery rate (FDR)–adjusted *P* values denoted by asterisks. (**F**) Expression of *Ppara* was induced in CD + PE or MD + PE groups relative to CD + Veh. (**G**) Expression of *Nr1i3* (also known as CAR) was induced in MD + PE groups relative to CD + Veh. (**H** to **J**) GSEA of NR-regulated lipid metabolism and toxicity gene set comparing CD + PE versus CD + Veh (H), MD + PE versus MD + Veh (I), and MD + PE versus CD + Veh (J). (**K**) Heatmap with the pheatmap package illustrating NR expression across treatment groups, with color reflecting relative expression and dot size indicating normalized counts. For each condition, the row means of normalized counts were calculated and transformed using log_2_ (mean + 1). The resulting values were then scaled across rows. Data are presented as the means ± SD. For all comparative analyses, significance was determined using DESeq2-derived FDR-adjusted *P* values. *FDR <0.05, **FDR < 0.01, and ***FDR < 0.001. ES, enrichment score; NES, normalized enrichment score.

Given that PE administration induced hepatotoxicity in mice, bulk RNA-seq was performed to understand the overall effect of experimental treatments (MD or/and PE) on hepatic gene expression. The principal components analysis (PCA) plot revealed the distinct transcriptomic profiles among the experimental groups (Fig. 2A). Differentially expressed gene (DEG) analysis identified 600 and 1437 genes uniquely altered by PE and MD treatment, respectively. In addition, 279 genes were differentially expressed in the MD + PE group compared to the MD group (Fig. 2B). Furthermore, when examining PE-specific DEGs under different dietary conditions, we found 503 genes uniquely altered by PE in the CD group, 182 genes in the MD group, and 97 genes shared between both conditions (Fig. 2C and tables S5 to S7). In the CD-specific group, down-regulated genes by PE included *Angptl8*, *Acp3*, *Lepr*, and *Hmgcr*, while *Cyp4a12a*, *Cyp26b1*, *Gadd45g*, and *Prkcb* were up-regulated (fig. S2, A and B). Among these overlapping DEGs, lipid metabolism regulatory genes were shown to be significantly affected, with up-regulation (e.g., *Plin4*, *mt-Nd6*, *Cyb5a*, and *Fubp3*) and down-regulation (e.g., *Pnpla3*, *Acly*, *Me1*, and *Fasn*), further highlighting PE’s role in transcriptional dysregulation in the liver (Fig. 2D). A combined effect of MD and PE exposure was also observed, highlighting dysregulation of cholesterol production (e.g., *Tm7sf2*, *Ldlr*, and *Mylip*), metabolic function and support (e.g., *Pask* and *Oit3*), and inflammation (e.g., *Nkap*) (Fig. 2E). Notably, MD and PE exposure significantly stimulated the expression of *Ppara* and *Nr1i3*, two well-established master regulators of toxicological and xenobiotic responses, respectively (Fig. 2, F and G). These findings reinforce the role of these NRs in orchestrating the liver’s response to the insults of an MD and PE particles. Given the critical role of NRs in regulating metabolic, inflammatory, and damage response pathways in the liver, we investigated how NR expression changes across our experimental conditions. Gene set enrichment analysis (GSEA) was performed to examine correlation between NR and liver toxicity. This revealed that not only in MD + Veh versus CD + Veh (fig. S2C) but also enrichment patterns induced by PE in both CD and MD mouse groups are closely linked to lipid metabolism regulation and hepatic toxicity (Fig. 2, H and I). Consequently, the MD + PE group exhibited the most pronounced induction of these toxicity-related pathways, reflecting a compounded exacerbation of hepatic stress beyond the level of the CD + Veh control (Fig. 2J). Furthermore, the heatmap revealed that a considerable shift of NR expression was observed in the treatment mouse liver compared to the control group at both the condition and sample levels (Fig. 2K and table S8). Notably, this correlation was more prominent in the MD group, indicating a potential synergistic effect of MD and PE on NR expression dysregulation and liver function (Fig. 2H and fig. S2, D and E). We also conducted a comparative analysis between liver transcriptomic data from PS-exposed mice (GSE245069) and our PE dataset. In doing so, several NRs exhibited consistent expression changes across both datasets, notably the up-regulation of *Ppara* and down-regulation of *Esr* (fig. S2, F and G). These results reveal that MPs, despite their varied types, can orchestrate both shared and unique influences on NR expression, underscoring the complex interplay between MP exposures and gene regulation.

### Spatial single-cell transcriptomic profiling of MD and PE effects on liver tissue

**Fig. 3.**
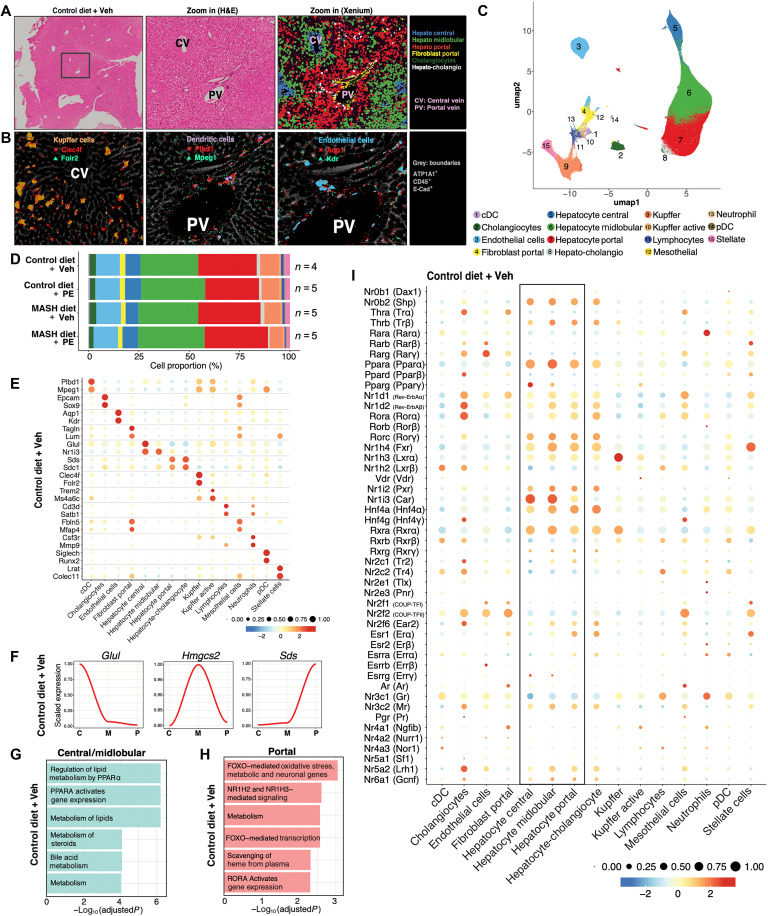
Spatial transcriptomics revealed distinct liver zonation and gene expression. (**A**) Overview of histological image, spatial single-cell membrane segmentation, and annotation of CD mouse liver using Xenium. (**B**) Spatial localization of stromal liver components using the Xenium Explorer: Kupffer cells (*Clec4f* and *Folr2*), dendritic cells (*Plbd1* and *Mpeg1*), and endothelial cells (*Aqp1* and *Kdr*). (**C**) Spatial transcriptomic data were transformed to the single-cell level using Uniform Manifold Approximation and Projection (UMAP) for dimensionality reduction, followed by cell type annotation based on marker gene expression of all conditions. Cell type labels include abbreviations as follows: cDC, conventional dendritic cells; hepato-cholangio, intermediate cells with features of both hepatocytes and cholangiocytes; pDC, plasmacytoid dendritic cells. (**D**) Average cell type proportions across experimental groups. The same color code for cell types as in Fig. 3C were applied. (**E**) A dot plot showing marker gene expression with two representing markers per cell type in the CD with vehicle group; color indicates relative gene expression levels, and dot size reflects the proportion of cells expressing each marker. (**F**) Validation of hepatocyte zonation markers: *Glul* (central, C), *Hmgcs2* (midlobular, M), and *Sds* (portal, P). (**G** and **H**) Enriched pathways in genes up-regulated in central/midlobular (G) and portal (H) hepatocytes from the control group. The Reactome database via Enrichr was used. (**I**) Expression profiles of NR genes across identified cell types in mice fed with CD only. Color indicates relative gene expression levels, while dot size reflects the proportion of cells expressing each gene. CV, central vein; PV, portal vein.

We conducted spatial single-cell profiling of RNA expression at the cellular and subcellular level using the Xenium platform to further delineate the intricate spatial dynamics between parenchymal and nonparenchymal cells within the liver (Fig. 3, A and B). On the basis of well-established cell markers, 15 different cell clusters were determined, and the spatial distribution and relative proportion of each cluster were subsequently observed and visualized (Fig. 3, C to E). Cell proportion analysis indicated a slight shift within the hepatic cellular compartment across experimental groups (Fig. 3D and fig. S3A). Specifically, a notable increase in the proportion of midlobular hepatocytes was observed in the PE-treated groups with a beta regression test. While this provides an initial overview of the cellular landscape, the regional transcriptomic alterations and the underlying regulatory mechanisms are further explored in greater depth during the subsequent spatial and mechanistic analyses. Of note, RNA profiling via Xenium was sensitive enough to resolve Kupffer cells into distinct *Trem2^+^* (active) and *Trem2^−^* (inactive) subsets in the control group (Fig. 3E). In addition, hepatocytes from the control group were characterized using zonation-specific markers, *Sds* for the periportal zone, *Hmgcs2* for the midlobular zone, and *Glul* for the pericentral zone (Fig. 3F and table S9). Other genes similarly displayed zonation-specific patterns (fig. S3B). To further validate the distinct biology of annotated liver zones, pathway analysis indicated zone-specific established biological features accordingly, e.g., lipid genesis features within central/midlobular hepatocytes (Fig. 3G), while bile acid homeostasis–related processes were enriched in portal hepatocytes (Fig. 3H). Collectively, profiling the expression of 48 NRs in the control mouse group across 15 hepatic cell types revealed distinct expression patterns, highlighting their role in orchestrating gene expression programs (Fig. 3I).

### PE MPs induce a marked shift in hepatocyte NR expression

**Fig. 4.**
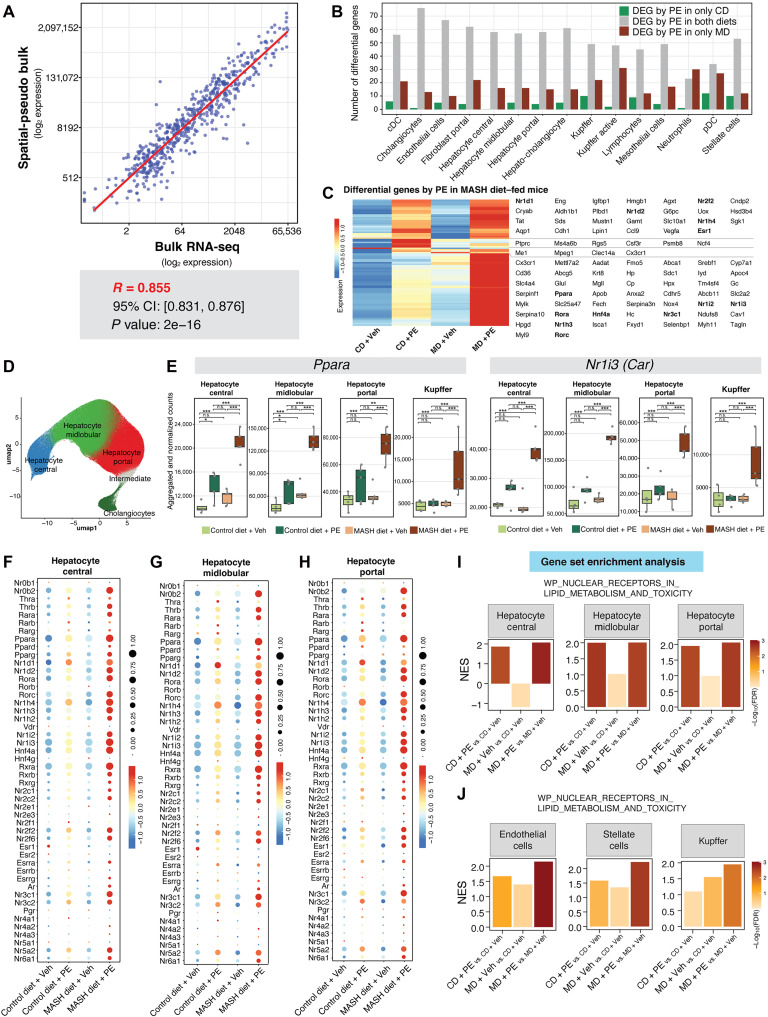
Spatial transcriptome single-cell data reveal differential gene expression and metabolic alterations in liver. (**A**) Pearson correlation analysis was performed between the spatial pseudo-bulk and bulk RNA-seq data. For the scatterplot visualization, log_2_ scale transformation was performed. (**B**) Number of DEGs (|log_2_ FC| ≥ 0.38, FDR < 0.05, min. expressing cell proportion ≥ 0.33) associated with PE treatment across various cell types and dietary conditions. (**C**) The heatmap shows MD-specific PE-induced genes, excluding those affected under the CD condition. Bolded entries highlight prioritized NRs and candidates for downstream analysis. (**D**) Single-cell level representation of spatial transcriptomic data using UMAP, illustrating subtypes of hepatocytes and cholangiocytes. (**E**) Distinct expression patterns of *Ppara* and *Nr1i3* based on sample-level aggregated and normalized counts across dietary and PE treatment groups in hepatocytes and cholangiocytes. Boxplots with the *ggplot2* package represent median, IQR, and 1.5× IQR whiskers. Pairwise differential expression was performed via *DESeq2*. (**F** to **H**) Comparison of NR gene expression across treatment groups within central (F), midlobular (G), and portal (H) hepatocytes; color indicates relative gene expression levels, while dot size reflects the proportion of cells expressing each gene. (**I** and **J**) GSEA of the NR lipid metabolism and toxicity gene set across three comparisons: CD + PE versus CD + Veh; MD + Veh versus CD + Veh; and MD + PE versus MD + Veh, performed in hepatocyte subtypes (I) and other major cell types (endothelial cells, stellate cells, and Kupffer cells) (J). Data are presented as the means ± SD. For all comparative analyses, significance was determined using FDR-adjusted *P* value. *FDR < 0.05, **FDR < 0.01, and ***FDR < 0.001. 95% CI, 95% confidence interval. min., minimum.

We then examined spatial pseudo-bulk gene expression and found a strong positive correlation with bulk RNA-seq expression from the matched mouse samples. This demonstrates that Xenium profile outcome is consistent with bulk sequencing results while retaining subcellular resolution (Fig. 4A). In line with bulk-RNA analysis, DEGs were identified among the CD + PE and MD + PE groups across each of the 15 cell types (Fig. 4B and tables S10 to S12). Of note, the number of DEGs within MD-fed groups was considerably higher compared to CD-fed groups (Fig. 4B). DEGs uniquely induced by PE under the MD condition, excluding those also altered by PE in CD, were identified across all cell types, highlighting key genes such as *Me1*, *Anxa2*, and *Ppara* (Fig. 4C). We next focused on hepatocytes, the predominant cell type in the liver, to explore condition-specific transcriptional changes and zonation-related patterns (Fig. 4D). In doing so, we found that the NR member genes *Ppara* and *Nr1i3* (CAR) exhibited distinct expression patterns across hepatocyte subclusters within each experimental group at the sample and cell levels (Fig. 4E and tables S10 to S12). *Ppara* and *Nr1i3* expression was markedly up-regulated in Kupffer cells specifically in the MD + PE condition (Fig. 4E and tables S10 to S12). In addition, we conducted comprehensive profiling of NR expression across individual cell type, uncovering distinct signatures that suggest the unique roles of NRs in mediating cell-specific responses to metabolic stress and PE exposure (Fig. 4, F to H; fig. S4, A to L; and tables S10 to S12). Then, GSEA was performed using the module, NR, and lipid metabolism and toxicity, to examine the effect of PE across cell types (Fig. 4, I and J, and fig. S4M). In doing so, significant enrichment was observed in the PE-exposed groups regardless of diet; however, no significant difference was seen between the CD- and MD-fed groups (Fig. 4I). Together, these findings may provide a potential molecular basis that PE acts as a driver of liver toxicity in hepatocytes. Similar trends were also observed in other cell types, with the highest enrichment seen under the combined effect of MD and PE (Fig. 4J).

### Spatial transcriptome profiling uncovers the relationship between the liver cellular composition and the degree of inflammation

**Fig. 5.**
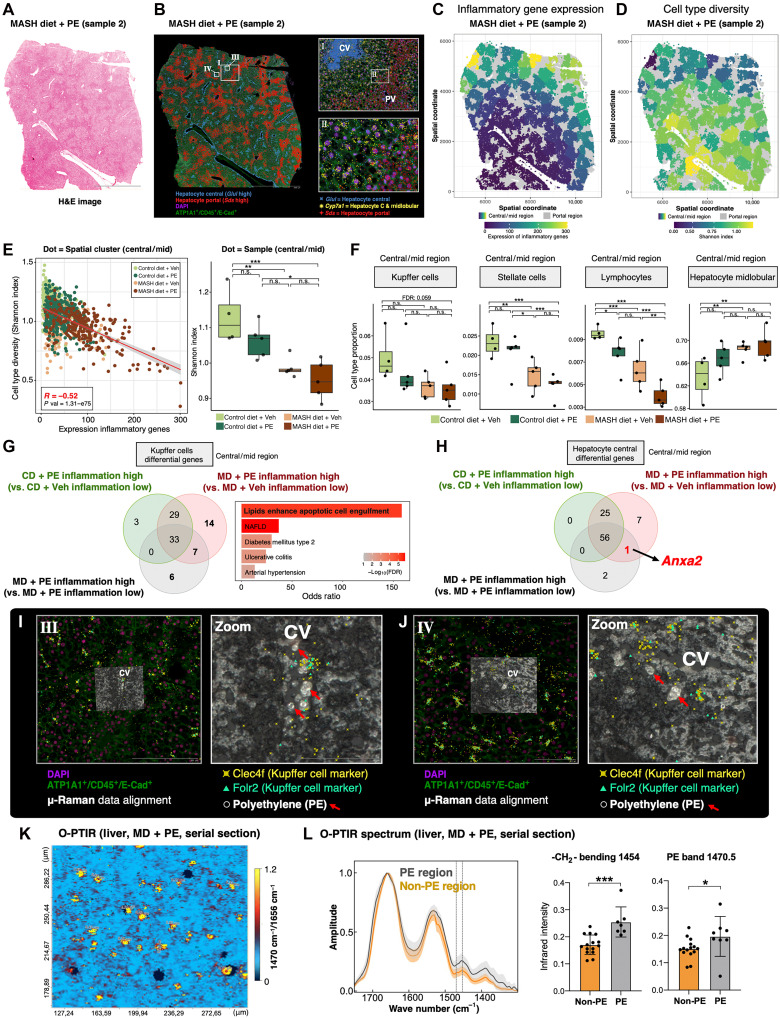
Spatial transcriptomics revealed inflammation and damage in MP-treated mice, showing altered cell diversity and gene expression. (**A** and **B**) Histological overview (A) and high-resolution spatial mapping of cell types (B) in a representative MD + PE liver sample. Central and portal hepatocyte enriched regions are overlaid with 4′,6-diamidino-2-phenylindole (DAPI) and boundary staining (green); zoomed regions (I and II) display CV and PV areas with zonation markers *Glul*, *Cyp7a1*, and *Sds*. (**C** and **D**) Identification of inflammatory regions in the MD + PE liver based on a GOBP-derived gene set (C) and measurement of cell diversity (D), within central/midlobular areas. (**E**) Inverse Pearson correlation between inflammatory gene expression and cell type diversity scores observed across central/midlobular regions from all conditions. Significance boxplot comparisons were assessed using linear regression followed by Tukey’s post hoc correction. (**F**) Cell type proportion shift across experimental groups in central/midlobular regions; significance was assessed using beta regression with Holm correction. (**G** and **H**) Venn diagram illustrating DEG overlaps among three comparisons in Kupffer cells (G) and central hepatocytes (H) from spatially clustered central/midlobular regions. Enrichment analysis (Enrichr) was performed on MD-specific DEGs using the Elsevier Pathway database. (**I** and **J**) Coregistration of Xenium data (regions III and IV) with Raman spectroscopy localizing PE-like particles (arrows) near Kupffer cell–enriched area (*Clec4f* and *Folr2*). (**K**) O-PTIR ratio imaging (1470/1656 cm^−1^) highlighting regions enriched in PE-associated -CH_2_- bending relative to tissue amide I signal in the liver. (**L**) Raman spectra of PE versus non-PE regions in the liver. Band enrichment (1454 and 1470 cm^−1^) was evaluated using the Mann-Whitney nonparametric test. Data are presented as means ± SD. Statistical significance: *P < 0.05, **P < 0.01, and ***P < 0.001 [FDR-adjusted *P* value for (E) and (F)]. NAFLD, nonalcoholic fatty liver disease.

In addition to conventional hematoxylin and eosin (H&E) staining (Fig. 5A), spatial transcriptome mapping enabled us not only to identify overall pathological signature activity but also to precisely trace signature hotspots within their original tissue configuration while maintaining subcellular resolution. For example, *Glul* for pericentral, *Cyp7a1* for midlobular, *Sds* for periportal within the MD + PE liver sample were examined (Fig. 5B). To further investigate PE-related liver damage, we interrogated the expression of C3, a gene associated with inflammation, and observed increased expression in the CD + PE, MD + Veh, and MD + PE groups compared to CD + Veh (fig. S5A). We further confirmed the enriched expression of *C3* in specific regions of MD + PE samples (fig. S5, B to D). Then, we focused on the central/midlobular regions of each sample, considering their increased vulnerability to injury within the liver, which was profiled through a surrogate inflammatory gene signature (i.e., *Alox5ap*, *C3*, *Dnase1l3*, *F3*, *Hp*, *Nupr1*, *Reg3g*, *Ccr7*, and *Il6*) across all samples. Accordingly, our binning- and clustering-based spatial analysis identified high- and low-inflammatory regions within each condition sample (Fig. 5C and fig. S5, E and G). In addition, we further analyzed cellular diversity using the Shannon index to determine how each treatment affected liver cell population dynamics, specifically within the pericentral and midlobular zones (Fig. 5D and fig. S5, F and H). Notably, we observed an inverse correlation (*R* = −0.52) between the inflammation gene signature expression and cellular diversity within MD + PE–treated liver samples and consistently across all experimental groups (Fig. 5E). Diversity alterations within individual cell types were analyzed on a sample basis (Fig. 5E and table S13). Statistical analysis revealed nonparenchymal liver cell proportions, including Kupffer and stellate cells and lymphocytes, significantly decreased in MD + PE–treated mice compared to CD + Veh (Fig. 5F and table S13). In contrast, the parenchymal cell proportion of midlobular hepatocytes remained slightly increased in the MD + PE group on a sample basis (Fig. 5F and table S13). Next, we examined Kupffer cells, considering their prominent role as the first damage responder in the liver. We found that functional activity–related genes in the Kupffer cells were also affected by MD and PE treatments. DEGs in spatially clustered Kupffer cells, localized to the pericentral/midlobular zones, were identified by three comparisons: (i) CD + PE_inflammation^high^ versus CD + Veh_inflammation^basal^, (ii) MD + PE_inflammation^high^ versus MD + Veh_inflammation^basal^, and (iii) MD + PE_inflammation^high^ versus MD + PE_inflammation^basal^ (Fig. 5G). As a result, 28 genes specific to the MD + PE condition were identified, and these were significantly associated with the lipid-induced apoptotic cell engulfment (Fig. 5G and table S14), suggesting active participation of Kupffer cells in the damaged/apoptotic cell removals. Given that the pericentral region has increased susceptibility to damage, we applied the same previously mentioned analytical approach. In doing so, we identified 10 genes specific to the MD + PE group (Fig. 5H). *Anxa2* was uniquely enriched in the high-inflammation regions of MD + PE–treated samples but only in pericentral hepatocytes (Fig. 5H and table S15). In contrast, midlobular and periportal hepatocytes shared commonly enriched genes including *Slc4a4* (fig. S5, I and J, and tables S16 and S17). To spatially detect PE MPs in liver tissue, we applied two orthogonal approaches: micro-Raman and optical photothermal infrared (O-PTIR) spectroscopy. Raman analysis identified PE particles on the existing Xenium slide from MD + PE samples but not in MD + Veh controls (fig. S5, K and L). Integration with Xenium spatial transcriptomic maps enabled spatial correlation of PE localization with exposure-responsive transcriptional programs, including proximity to Kupffer cells in PE-positive regions, III and IV from Fig. 5B (Fig. 5, I and J). O-PTIR analysis of serial sections independently confirmed the PE presence by detecting its characteristic chemical signatures within the MD + PE tissues (Fig. 5, K and L). Together, we identified zone-specific inflammatory signatures across key hepatic cell types, including Kupffer cells and hepatocytes, in response to MASH-inducing diet and/or PE treatment.

### *Anxa2* expression in pericentral hepatocytes is correlated with *Ppara*

**Fig. 6.**
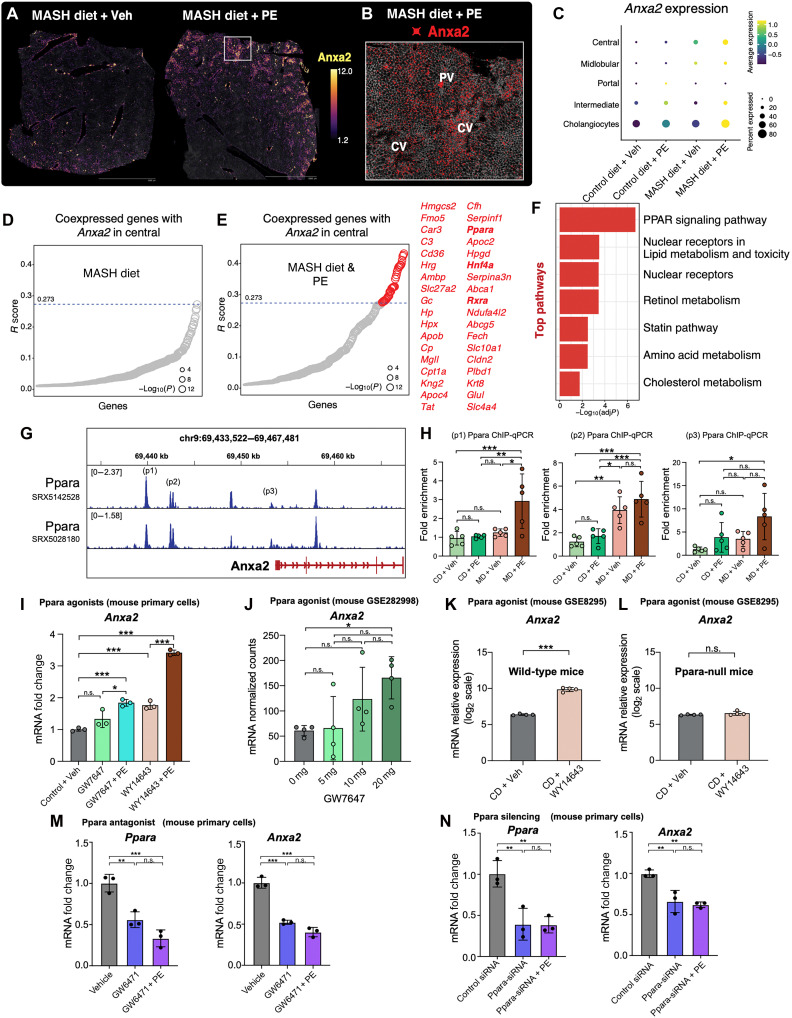
Ppara modulates *Anxa2* expression in the liver in response to MP and diet. (**A** and **B**) Density plot of *Anxa2* transcripts in MD + Veh and MD + PE samples (A). *Anxa2* density correlates with high-inflammatory regions near the CV and PV (B). (**C**) Dot plot illustrating the differential *Anxa2* expression among hepatocyte subtypes, intermediate cells, and cholangiocytes; color represents the average *Anxa2* expression, and dot size indicates the proportion of expressing cells. (**D** and **E**) Spearman correlation analysis of *Anxa2* with other mRNAs in central hepatocytes from MD + Veh samples, relative to the CD + Veh group (D). In MD + PE samples (E), genes with an *R* score > 0.273 [the maximum *R* score observed in (D)] are highlighted in red, indicating enhanced positive correlation. Circle size reflects −log_10_ transformed *P* values. (**F**) Enrichr analysis using the WikiPathways database identifying the PPAR signaling pathway as the top-ranked enriched term. (**G** and **H**) ChIP-seq (G) and ChIP-qPCR (H) validation of PPARα binding to *Anxa2* enhancer and promoter sites (peaks p1, p2, and p3). (**I** to **L**) In vitro (I) and in vivo [(J); GSE282998] induction of *Anxa2* by PPARα agonists (GW7647 and WY14643). The PPARα agonist, WY14643, induced *Anxa2* expression in vivo (K), while deletion of *Ppara* (null mice) abolishes agonist-induced *Anxa2* expression (L). (**M** and **N**) Loss-of-function assays: Pharmacological inhibition with the selective PPARα antagonist GW6471 (10 μM) and siRNA-mediated *Ppara* silencing both significantly repress PE-induced (100 μg/ml) *Anxa2* expression in primary mouse cells. Data are presented as mean ± SD. Statistical significance was determined using ordinary one-way ANOVA with Tukey’s multiple comparisons test or Spearman correlation analysis. *P < 0.05, **P < 0.01, and ***P < 0.001. FDR-adjusted for (D) to (F). ChIP-qPCR, chromatin immunoprecipitation quantitative polymerase chain reaction.

To further analyze whether *Anxa2* expression is specific to pericentral hepatocytes, its expression in parenchymal cells was investigated in the MD + PE_inflammation^high^ regions (Fig. 6, A and B). Notably, average *Anxa2* expression in central hepatocytes was also significantly higher in the MD + PE group compared to the MD + Veh group, suggesting a potential PE-specific modulatory effect (Fig. 6C). In contrast, the difference between CD + Veh and CD + PE groups was relatively modest (fig. S6A). Moreover, we validated significantly increased *Anxa2* expression in the MD + PE group by quantitative reverse transcription polymerase chain reaction RT-qPCR (fig. S6B). *Anxa2* was not only significantly increased in expression but also showed an increased proportion of pericentral hepatocytes expressing the gene in the MD + PE group compared to the CD + Veh group at the sample and cell levels (Fig. 6C and tables S10 to S12). A similar trend was observed in other cells, including midlobular hepatocytes, intermediate cells, and cholangiocytes, but not in portal hepatocytes (Fig. 6C and tables S10 to S12). To further delineate the specific effect of PE on *Anxa2* expression within central hepatocytes, we performed a correlation analysis between *Anxa2* and other genes within this subset across three comparisons: (i) CD + PE versus CD + Veh (fig. S6C), (ii) MD + Veh versus CD + Veh (Fig. 6D), and (iii) MD + PE versus CD + Veh (Fig. 6E). By setting the maximum *R*-score observed in MD effect groups as a threshold, shown as a broken line, genes exceeding this value were identified as potential positive correlates of Anxa2, specifically under the MD + PE condition (Fig. 6E). A total of 36 MD + PE condition–specific genes correlated with *Anxa2* were detected (Fig. 6E). PPAR signaling was identified as the top-ranked enriched pathway using the correlated genes (Fig. 6F). To independently validate these findings using an unsupervised approach, we performed sample-based weighted gene coexpression network analysis (WGCNA) on central hepatocytes. This analysis identified a distinct gene module (Blue module) significantly correlated with the MD + PE condition (*R* = 0.68, *P* = 0.001) (fig. S6D). Notably, *Ppara* emerged as a central hub gene, and functional enrichment analysis confirmed that PPAR signaling was the dominant pathway underlying the MD + PE–specific gene signature (fig. S6E). With this finding, we further examined two PPARα chromatin immunoprecipitation sequencing (ChIP-seq) datasets and found that its complex potentially binds to Anxa2 enhancer and promoter regions (Fig. 6G). To access these findings, ChIP-qPCR was performed using our experimental liver samples (Fig. 6H). We confirmed significant PPARα enrichment at the selected Anxa2 peaks (p1, p2, and p3) identified, as shown in Fig. 6G. To assess the induction of *Anxa2* as a downstream target of PPARα, mouse primary liver cells were treated with the PPARα agonists, GW7647 and WY14643, and compared to vehicle-treated controls. Both agonists elicited significant increase *Acox1* levels, a well-established PPARα target (fig. S6F). Then, we further confirmed that two agonists induced *Anxa2* levels relative to vehicle-treated controls, with WY14643 producing a more pronounced up-regulation than GW7647 (Fig. 6I). Moreover, cotreatment with PE further enhanced *Anxa2* expression. Collectively, these results indicate that activation of PPARα by these ligands up-regulates *Anxa2* expression. To further validate this association, we analyzed publicly available in vivo datasets of PPARα agonists, GW7647 and WY14643. The analysis from the GSE282998 revealed that the selective PPARα agonist, GW7647, increased *Anxa2* expression in a dose-dependent manner (Fig. 6J). Furthermore, we used the GSE8295 dataset and examined another PPARα-selective agonist, WY14643, in WT mice and *Ppara*-null mice (Fig. 6, K and L). In WT mice, treatment with WY14643 resulted in a significant increase in *Anxa2* expression (Fig. 6K). In contrast, in *Ppara*-null mice, the same WY14643 treatment failed to induce any significant change in *Anxa2* levels (Fig. 6L). The absence of a response in *Ppara*-null mice confirms that WY14643-induced *Anxa2* up-regulation is significantly dependent on PPARα, firmly establishing *Anxa2* as a downstream target of PPARα signaling. To further validate this mechanism in the context of PE exposure, loss-of-function studies in primary hepatocytes demonstrated that both pharmacologic inhibition with the PPARα antagonist GW6471 (Fig. 6M) and RNA silencing using *Ppara*–small interfering RNA (siRNA) (Fig. 6N) abolished PE-induced *Anxa2* up-regulation, establishing that this response is mediated through the PPARα pathway. Overall, these findings suggest that *Anxa2* expression in liver cells is modulated by PPARα.

## DISCUSSION

A growing body of research has demonstrated the harmful impact of MPs on liver health (*39*, *46*–*49*). Knowing the existence of MPs in cirrhotic human liver tissues, this suggests that chronic MP exposure may contribute to liver disease progression (*50*). Despite these valuable insights into MP-related hepatotoxicity, most studies predominantly focused on PS exposure. This leaves a critical knowledge gap, especially since PE is the most accumulative MP found in human liver (*45*).

In our study, in vivo exposure to PE micro-size particles induced significant hepatotoxicity in mice, markedly disrupting lipid metabolism. Notably, coadministration of PE particles with MD diet treatment further amplified these effects, leading to more severe liver damage and dysregulated NR signaling. Thus, we leveraged spatial transcriptomics (Xenium: 10X Genomics) to investigate intercellular interactions within the inflammatory region in vivo samples. Our spatial analysis focused on the central to midlobular zones, which were hypothesized to be the most severely affected by PE exposure. The central zone is particularly susceptible to injury due to its low oxygen supply, high metabolic activation of toxins, and early injury of sinusoidal endothelial and stellate cells (*46**,* *51**–**53*). This established the basis of our hypothesis that PE-mediated damage would also manifest severely in this region.

Spatial profiling enabled us to quantify cell type diversity, revealing that cellular diversity was inversely correlated with inflammation. Notably, MD alone caused a modest reduction in cell diversity, whereas MD + PE resulted in a markedly greater loss. This reduction in diversity was primarily attributed to an increased proportion of midlobular hepatocytes, accompanied by a marked decline in nonparenchymal cell populations such as Kupffer cells. Notably, the proportion of central hepatocytes remained largely unchanged, which was unexpected. Nevertheless, our hypothesis is supported by the PE-specific DEGs observed in Kupffer cells, which strongly suggest the presence of lipid-induced apoptotic cell engulfment activity within the damaged central and midlobular regions of the liver in MD + PE–treated mice. This phenomenon may be explained by the identification of PE-specific DEGs uniquely enriched in central hepatocytes, with Anxa2 emerging as a key candidate.

Previous studies have highlighted two distinct perspectives on the role of hepatocyte-specific *Anxa2* in liver injury. In diet-induced models, Anxa2 promotes fibrosis via activation of hepatic stellate cells, contributing to metabolic dysfunction-associated fatty liver disease (MAFLD) progression (*54*). Conversely, in acetaminophen (APAP)–induced injury, ANXA2-positive hepatocytes support tissue regeneration by migrating into necrotic zones and aid in tissue repair (*55**,* *56*). Our study demonstrated that *Anxa2* expression in hepatocytes was significantly elevated following MD + PE treatment, despite the absence of overt fibrosis. Given that *Anxa2* expression was not altered by high-fat diet alone in Wang *et al.*’s study (*54*), our results suggest that the up-regulation of *Anxa2* is additionally driven by PE exposure. This implies a distinct mechanism of PE-induced hepatocyte stress or early remodeling, potentially independent of traditional fibrogenic signaling pathways. Supporting this idea, we observed a significant increase in *Anxa2*-expressing central hepatocytes in MD + PE–treated samples (Fig. 6C). Similarly, Kupffer cells with engulfing activity were observed in the pericentral/midlobular region, supporting the notion of an early or partial regenerative response initiated by PE-induced hepatic stress. Together, these observations raise the possibility that Anxa2 marks a stress-responsive hepatocyte state capable of migrating or remodeling tissue even outside the context of APAP-induced toxicity.

Our study identified PPAR signaling as one of the most enriched pathways in *Anxa2* expressing central hepatocytes of MD + PE–treated mouse livers (Fig. 6F), suggesting a potential role in zone-specific transcriptional regulation during hepatic stress. PPARα activation may facilitate hepatic stress adaptation or regenerative programming via *Anxa2* up-regulation, particularly in central zone hepatocytes under PE exposure. Of note, this mechanistic insight aligns with a growing body of clinical research targeting PPARα for the treatment of liver diseases, including MASH. Multiple clinical trials have investigated PPARα-targeting compounds, such as pemafibrate, a selective PPARα modulator (*43*), and saroglitazar, a dual PPARα/γ agonist, both of which have demonstrated reductions in liver stiffness, transaminases, and steatosis in patients with MAFLD and patients with MASH (*42*). Moreover, lanifibranor, a pan-PPAR agonist (α/δ/γ), showed significant histological improvement in a phase 2b trial, including MASH resolution and fibrosis regression, and is now under evaluation in phase 3 trials (*44*). Our findings not only underscore the functional importance of PPARα signaling in liver homeostasis during PE exposure but also provide potential mechanistic insight into how the PPARα-ANXA2 axis might be modulated during hepatic injury at the hepatocellular level.

While this study provides high-resolution insights into the spatial molecular alterations induced by MD and PE exposure, several limitations warrant consideration. First, rigorous cellular segmentation in liver tissue remains technically challenging due to the complex, interdigitated morphology of hepatocytes and sinusoidal nonparenchymal cells. Although we used advanced algorithmic methods to define cellular boundaries, the possibility of minor signal spillover between adjacent cells cannot be entirely excluded. Second, the 10X Genomics Xenium platform uses a targeted high-resolution spatial transcriptomics approach. While our custom panel was comprehensively designed to capture key liver metabolism–related genes, this targeted strategy does not capture the full transcriptome. Consequently, the unbiased discovery of low-abundance transcripts or novel regulatory elements outside this predefined panel was beyond the scope of the current spatial analysis. Last, while our data strongly suggest the involvement of the PPARα-ANXA2 axis, this study primarily focuses on the mechanistic characterization of this pathway rather than evaluating PPARα modulation as a direct clinical therapeutic intervention.

Despite these limitations, this study represents one of the first applications of high-resolution spatial transcriptomics in the context of MP-induced liver toxicity. We demonstrated that PPARα directly regulates *Anxa2* expression, forming a regulatory axis that may contribute to hepatocyte adaptation or regenerative programming under metabolic or PE challenge. These findings not only expand our understanding of zone-specific transcriptional networks in liver injury but also provide mechanistic context that complements the growing number of clinical studies targeting PPARα for MASH and related liver diseases. Together, our results highlight the dual biological and translational significance of the PPARα-ANXA2 axis. Further studies are needed to identify the upstream cellular pathways that lead to PPARα activation in this context, as this will be essential for advancing PPARα- and ANXA2-targeted therapies and deepening our understanding of their roles in liver pathophysiology.

## MATERIALS AND METHODS

### Cell culture and in vitro treatments

Mouse primary hepatocytes were isolated from male C57BL/6 WT male mice using the collagenase perfusion methods as previously described (*57*). Isolated primary hepatocytes were plated in a 96-well plate at a density of 50,000 cells per well in William’s E medium containing penicillin (100 U/ml), streptomycin (100 μg/ml), and 5% fetal bovine serum (FBS). Hepatocytes were treated with MPs (100 μg/ml), namely, PE (CPMS-0.96, 10 to 150 μm, Cospheric), PVDC (CV30-PD-000110, 180 μm, Goodfellow), PET (ES30-PD-000132, 300 μm, Goodfellow), PP (PP30-GL-000112, 5000 μm, Goodfellow), PA-6 (AM30-PD-000155, 55 μm, Goodfellow), PS (PSMS-1.07, 85 to 105 μm, Cospheric), and PVC (81388, Sigma-Aldrich) for 24 hours. For the PPARα agonist and antagonist study, mouse primary hepatocytes were isolated from male C57BL/6 WT male mice using the collagenase perfusion method. Cells were plated in a six-well plate with a density of 500,000 cells per well in William’s E medium containing penicillin (100 U/ml), streptomycin (100 μg/ml), and 5% FBS. For PPARα agonist and antagonist treatments, hepatocytes were treated with the following agonists: 10 μM GW7647 or 10 μM WY14643 (agonists) or 10 μM GW6471 (antagonist) or dimethyl sulfoxide (DMSO; vehicle) for 24 hours. For agonist/antagonist + PE group, hepatocytes were concomitantly treated with PE (100 μg/ml) in DMSO for 24 hours. For in vitro knockdown studies, hepatocytes were transfected with 1 μg ON-TARGETplus Mouse *Ppara* (19013) siRNA-SMARTpool (#L-040740-01-0005) and nontargeting siRNA (#D-001810-10-05) (Horizon Discovery, Boyertown, PA) using METAFECTENE FluoR transfection reagent (#T050, Biontex, Munich, Germany) according to the manufacturer’s instructions. Twenty-four hours after transfection, cells were treated with vehicle and PE (100 μM) for an additional 24 hours.

### MTT assay

Plated mouse primary hepatocytes were rinsed twice with William’s E medium, and 100 μl of thiazolyl blue tetrazolium bromide (MTT) reagent (Thermo Fisher Scientific) (0.5 mg/ml) was added to each well. After 5 hours of incubation at 37°C, 100 μl of solubilization buffer (DMSO:radioimmunoprecipitation assay, 50:50) was added to each well. After overnight incubation at 37°C, the absorbance was measured at 570 nm using a Synergy ultraviolet-visible multiwell plate spectrophotometer (Agilent Technologies, Santa Clara, CA). Raw values were presented in table S1.

### Animals and in vivo treatments

Eight-week-old male C57BL/6 were procured from the Jackson Laboratory (Bar Harbor, ME) and used per the guidelines of the Institutional Animal Care and Use Committee at the University of Oklahoma Health Sciences Center (23-056-SEAFHI). The mice were accommodated in environmentally controlled cages, ensuring a constant temperature of 22° ± 1°C, a relative humidity of 25 ± 5%, and a standard 12-hour light/dark cycle. Groups of mice (*n* = 4 or 5 per group) were housed together and provided free access to food and water. Mice were given either a high-fat, high-fructose, high-cholesterol diet termed MD (40 kcal% fat, 20 kcal% fructose, and 2% cholesterol) (#D09100310, Research Diets, New Brunswick, New Jersey) or a CD (10 kcal% fat and matching sucrose) (#D09100304, Research Diets) ad libitum for 8 weeks. For the mice treated with MP (CD + PE and MD + PE groups), 2 mg PE (10 to 150 μm in diameter, Cospheric, Somis, CA) suspended in 0.1% Tween 20 + 99.9% molecular-grade water was administered by oral gavage per mouse per day for 8 weeks. For the vehicle groups (CD and MP groups), mice were orally gavaged daily with 0.1% Tween 20 of 99.9% molecular-grade water without PE. Body weight and food intake were measured weekly. At the end of the 8-week study, mice were euthanized by inhalant anesthetic overdose of isoflurane, followed by removal of vital organs as a secondary assurance method.

### Histology

Liver tissues were fixed in 10% neutral buffered formalin and submitted to the Tissue Pathology Core at the Stephenson Cancer Center, University of Oklahoma Health Sciences Center, for paraffin embedding, sectioning (5 μm), and H&E staining. Images were captured on the Echo Revolve microscope (Echo, San Diego, CA). Histopathological analysis, including nonalcoholic fatty liver disease activity scoring, was performed in a blinded manner.

### Hepatic TG and liver injury measurements

A Triglyceride-Glo (#J3160, Promega, Madison, WI) assay kit (#J3190, Promega) was used to quantitate triacylglycerol content from mouse livers. ALT assay activity in serum was measured fluorometrically using an ALT activity assay kit (#ab105134, Abcam, Cambridge, MA) according to the manufacturer’s protocol.

### Bulk RNA-seq and analysis

RNA was extracted using TRIzol (#15596018, Thermo Fisher Scientific, Waltham, MA). Then, RNA quality was checked using the Agilent’s 2100 Bioanalyzer. Stranded RNA-seq libraries were constructed using an NEBNext poly(A) mRNA isolation kit followed directly by the Integrated DNA Technologies’ (IDT) XGen Broad Range RNA Library Prep Kit and the established protocols. The library construction was done using up to 1 μg of RNA. Each of the libraries was indexed during library construction to multiplex for sequencing. Libraries were quantified using the Invitrogen’s Qubit 4 fluorometer and checked for size and quality on the Agilent’s 2100 Bioanalyzer. Samples were normalized and pooled onto a 150 paired end run on the Illumina’s NextSeq 2000 Platform to obtain 15 million reads per sample at the Genomics Core at the University of Oklahoma Health Campus (OUHC).

Raw RNA-seq reads were preprocessed for quality control using fastp (v0.23.4) with adapter trimming, poly-G, and poly-X removal. Following quality control (QC), filtered paired-end RNA-seq reads were quantified using Salmon (v1.10.3) with selective alignment mode enabled (--validateMapping), allowing more accurate mapping of reads to the GRCm39 transcriptome from the Encyclopedia of Genes and Gene Variants (GENCODE). Library type was automatically detected (−l A), and sequence bias correction (--seqBias) and variational Bayesian optimization (--useVBOpt) were applied. Resulting quantification files were collected for downstream analysis. Gene-level abundance estimates were imported into R using the tximport package, using a transcript-to-gene mapping file. Differential expression analysis was performed using DESeq2. After estimateSizeFactors were calculated, the DESeq function was used. The analysis included multiple condition comparisons, and the final DEG results were exported for downstream interpretation. For differential gene expression analysis, a log_2_ fold change (FC) cutoff of ±0.38 and a false discovery rate (FDR) threshold of less than 0.05 were applied. To explore variance within bulk RNA-seq data within samples, dimensionality reduction was done using PCA, which was conducted using the plotPCA function. For heatmap generation, with NR expression, the pheatmap package was used. Expression values were transformed to log_2_ scale by computing logw of meanCounts + 1. GSEA was performed using the fgsea package in R. Ranked gene statistics were used as input, and enrichment was calculated against predefined gene sets with a minimum gene set size of 1 and a maximum of 500. A total of 10,000 permutations were conducted to estimate significance. Enrichment plots for selected module, LIPID_METABOLISM_AND_TOXICITY, were generated using the plotEnrichment function and visualized with ggplot2-based customization. An additional publicly available bulk RNA-seq dataset from PS-treated mouse liver (GSE245069) was retrieved, processed, and analyzed using the similar pipeline for the current study’s bulk RNA-seq data.

### Xenium for formalin-fixed paraffin-embedded samples

Formalin-fixed paraffin-embedded tissue samples were prepared, sectioned (5 μm), and embedded on Xenium slides by Tissue Pathology Core at the Stephenson Cancer Center, University of Oklahoma Health Sciences Center. Slides were then processed for the deparaffinization and decrosslinking procedure as outlined in 10X Genomics protocol (#CG000580). Afterward, Xenium In Situ Gene Expression preparation (probe hybridization, ligation and amplification, and nuclei staining) was performed following the protocol (#CG000749). Spatial profiling was achieved with tissue sections using the Xenium analyzer with the protocol (#CG000584). Post-Xenium H&E staining was completed by following the 10X demonstrated protocol (#CG000613) to achieve high-resolution visualization of tissue morphology.

### Xenium gene profiling

The 10X Genomics Mouse Tissue Atlassing gene panel, comprising 379 gene markers for various tissues and cell types, was used with an additional 100-gene panel consisting mainly of established NR genes as well as liver-related genes (table S2). Xenium Ranger output data for each slide run were retrieved using default parameters for downstream analysis.

### Spatial transcriptomic data processing and analysis

The processed data were used for downstream analysis by Seurat (v5.2.0). Xenium output including centroid and segmentation polygon was loaded into Seurat environment. A total of 19 samples from two Xenium slides were integrated, and layers were joined using the JoinLayers command. For quality control, low-quality cells with less than 25 unique transcripts were filtered out. After merging data from four treatment groups including vehicle-gavaged in CD-fed mice (CD + Veh), PE-gavaged in CD-fed mice (CD + PE), vehicle-gavaged in MD-fed mice (MD + Veh), and PE-gavaged in MD-fed mice (MD + PE), normalization was performed using single-cell transformation (SCT). Then, variable feature genes were identified using the FindVariableFeatures function, followed by data scaling with the scaledata command. PCA with Seurat RunPCA function was used. The Harmony package was used to account for batch correction across experimental groups and samples in combined data. Accordingly, the harmony-reduced embeddings were used to generate a Uniform Manifold Approximation and Projection (UMAP) with the RunUMAP function with 50 neighbors and a minimum distance of 0.001 and a spread of 1. Downstream graph-based clustering was done using shared nearest neighbor graph construction, and clusters were identified using the FindClusters function with a resolution of 0.8 resulting in 32 clusters. To identify cluster-specific marker genes, differential expression analysis was done using the FindAllMarkers function, and only positive markers were considered with minimum expression at least 25% of cells within the cluster and a log FC threshold of 0.25. The identified marker genes were used to manually annotate clusters by comparison with a reference liver single-cell atlas, and canonical marker genes (e.g., hepatocyte subtypes, cholangiocytes, Kupffer cells, endothelial cells, stellate cells, and immune cells) were used to identify major cell types. Cells with multiple heterogeneous expression patterns of mixed lineage signatures were flagged as low-quality populations and excluded from downstream analyses. The number of DEGs associated with PE treatment was assessed across various cell types and dietary conditions. Within each cell type, DEGs were identified using a log_2_ FC threshold of ±0.38, a minimum expressing cell proportion of 0.33, and an FDR of less than 0.05. Cell type proportions across experimental groups including CD + Veh, CD + PE, MD + Veh, and MD + PE were averaged from four to five biological replicates. Marker gene expression and the proportion of expressing cells were visualized with dot plots, where dot color reflected the expression level and dot size represented the percentage of expressing cells. UMAP, proportion, and dot plot were generated using the ShinyCell package. For line plot generation from hepatocyte subtypes, normalized gene expression data for selected genes of interest were visualized across liver zonation groups using ggplot2 in R. Expression values were plotted with smoothed locally estimated scatterplot smoothing (LOESS) curves to illustrate trends across anatomical zones. The geom_smooth function was used for pattern illustration. Then, genes up-regulated in zonally defined hepatocytes from control group were subjected to pathway enrichment analysis using the Reactome database via Enrichr. To compare matched spatial transcriptomic and bulk RNA-seq data, spatial data were pseudo-bulked by summing raw gene expression counts across all segmented cells within each spatial capture area. Corresponding bulk RNA-seq profiles were generated from matched liver tissues. Mean expression values of overlapping genes were log_2_-transformed and used for Pearson correlation analysis to assess concordance between the CD + Veh and MD + PE groups. GSEA was performed using the fgsea package on spatial transcriptomic data, focusing on a curated gene module related to lipid metabolism and toxicity. Gene expression was aggregated using the AggregateExpression function, followed by normalization with DESeq2. Comparisons included CD + PE versus CD + Veh, MD + Veh versus CD + Veh, and MD + PE versus MD + Veh. GSEA was conducted separately for hepatocyte subtypes and other major liver cell populations. Normalized enrichment scores and FDR were calculated to assess the direction and significance of enrichment. To examine molecular alterations like inflammation spatially, Xenium data were transformed into binned data, and clustering was performed on hepatocyte-enriched regions, focusing on hepatocytes adjacent to central-midlobular regions and those adjacent to portal veins. Bins preclassified regions were extracted for separate clustering analysis. Each initial K-means cluster was subsequently refined using the DBSCAN package with a neighborhood radius of 250 μm and a minimum of 10 points to define a dense cluster. This step enabled identification of more localized, tightly packed hepatocyte clusters, capturing spatial variations in cell density. Last, the average expression of specific gene sets was quantified within these identified hepatocyte clusters. This included individual genes like *Anxa2* and *Ppara*, as well as a panel of inflammation-related genes such as *Alox5ap*, *C3*, *Dnase1l3*, *F3*, *Hp*, *Nupr1*, *Reg3g*, *Ccr7*, and *Il6*. To assess cellular heterogeneity, the proportion of different cell types within each central-midlobular cluster or portal regions was determined. Cell counts for each type were aggregated per cluster and then divided by the total number of cells in that cluster to yield proportions. Subsequently, the Shannon diversity index was calculated for each cluster based on these cell type proportions, providing a quantitative measure of cellular heterogeneity. Correlation analysis was conducted to examine the relationship between gene expression and cell type diversity within central-midlobular or portal clusters. From each sample data, expression levels of immune and stress-related genes were extracted, and the Shannon index was extracted accordingly. Pearson correlation coefficients were computed between gene expression values and the Shannon index. Correlation results were visualized using scatterplots using the ggplot2 package with the geom_smooth option. Boxplots were generated to illustrate changes in the proportion of cell types across all four conditions, based on data from central and midlobular regions. Each boxplot displays the median as a horizontal line within the box, with the interquartile range extending from the first to the third quartile. Whiskers represent values within 1.5 times the interquartile range from the quartiles. For statistics, ordinary one-way analysis of variance (ANOVA) with Tukey’s post hoc correction was tested using the Prism. Using clustered information that included inflammation scores, spatial clusters were categorized into inflammation-basal, inflammation-middle, and inflammation-high groups. This classification was based on the distribution of inflammation scores across all clusters from all conditions, using the 0.33 and 0.67 quantiles as thresholds for stratification. To identify DEGs across (spatially examined) inflammatory conditions and groups, biologically relevant comparisons were defined using combinations of group, spatial region, and inflammation level. After extracting each of 15 cell types including hepatocyte subtypes, endothelial cells, Kupffer cells, and stellate cells, DEGs were further identified on the basis of conditions using the FindMarkers command. Then, using a log_2_ FC threshold of ±0.58, a minimum expressing cell proportion of 0.25, and an FDR of less than 0.05. Pathway enrichment analysis was performed using Enrichr with the Elsevier Pathway database on 28 DEGs identified in the MD group, excluding DEG derived from CD groups. To identify *Anxa2*-assoicated genes, central hepatocytes were subset and filtered by experimental groups. Normalized expression data from SCT assay were extracted, and gene-wise Spearman correlations were computed against *Anxa2* expression. Correlation coefficients (*R*) and *P* values were calculated for each gene. Significantly correlated genes were visualized with circle plots, where circle size reflects −log_10_(*P* value), and genes with *R* > 0.273 in MD + PE were highlighted.

### Statistical analysis of diversity metrics and cell type proportions

#### Sample-based analysis

To assess the overall complexity of the cellular ecosystem, the Shannon diversity index was calculated for each spatial cluster using the vegan R package. To avoid pseudoreplication, cluster-level indices were averaged to the biological sample level, and the mean Shannon index per sample was used for downstream analysis. Differences among the four experimental groups were evaluated using one-way ANOVA implemented as a linear model, followed by Tukey’s post hoc test for pairwise comparisons with the rstatix package. For cell type proportions, which are bounded within the (0, 1) interval, we used beta regression using the betareg package. Pairwise comparisons of marginal means were conducted using the emmeans package, with *P* values adjusted using the Holm-Bonferroni method to control the family-wise error rate.

#### Spatial cluster-based analysis

For cluster-level diversity analysis, Shannon diversity indices were analyzed using linear mixed-effects models implemented in the lme4 package, with biological sample included as a random intercept to account for intrasample correlation [Shannon ~ condition + (1 | sample)]. Cluster-level cell type proportions were analyzed using beta generalized linear mixed-effects models implemented in the glmmTMB package, including biological sample as a random intercept [Proportion ~ condition + (1 | sample)]. Pairwise comparisons were performed using estimated marginal means calculated with the emmeans package. *P* values were adjusted for multiple testing using Tukey correction for Shannon diversity models and Holm adjustment for beta models.

### Weighted gene coexpression network analysis

WGCNA was performed to identify gene coexpression modules within hepatocyte central cells. Cells were subset from the integrated Seurat object, and a pseudo-bulk approach was applied by averaging gene expression counts per biological sample using AverageExpression function, generating a gene-by-sample matrix. The matrix was generated, and low-quality genes were filtered using goodSamplesGenes. A soft-thresholding power was selected using pickSoftThreshold based on scale-free topology criteria (signed *R*^2^ ≥ 0.85); power 8 was chosen. Signed networks were constructed using blockwiseModules (power = 8, TOMType = signed, minModuleSize = 15, mergeCutHeight = 0.25). Module eigengenes were calculated as the first principal component of each module. Experimental groups were encoded as binary traits at the sample level. Module-trait associations were assessed using Pearson correlation, with *P* values computed using Student’s asymptotic test. Correlation coefficients and *P* values were visualized using labeled heatmaps. The module membership and trait association of *Ppara* were specifically examined.

### Raman spectroscopy

Raman analyses were performed using a micro-Raman spectrometer (XploRA Plus, HORIBA Scientific, Japan) with the existing Xenium performed slides. Initial optical imaging was conducted to assess the overall distribution of particles on the sample surface. Large-area overview images were acquired at ×5 and ×20 magnifications, and extended surface regions were reconstructed using the mosaic imaging function to generate representative images covering broad sample areas. On the basis of these overview images, individual particles of interest were identified and subsequently examined at higher magnifications (×50 and ×100) to enable detailed morphological characterization before spectral acquisition. Raman spectra were collected using a 532-nm excitation laser (maximum output power of 100 mW) equipped with a 1200 grooves mm^−1^ grating (750 nm). PE spectral acquisition was performed over a Raman shift range of ~1745 to 3440 cm^−1^, encompassing characteristic vibrational modes commonly used for polymer identification. Spectra were acquired with an integration time of 1 s and 32 accumulations to improve the signal-to-noise ratio. To minimize laser-induced heating or particle degradation, laser power was carefully adjusted and maintained at either 1 or 10% of the maximum output, depending on particle size and observed thermal stability. These acquisition parameters were selected to ensure high-quality spectra while preventing thermal damage during measurement. To minimize contamination, samples were handled using nonplastic tools where feasible and kept covered during transport and analysis.

### O-PTIR spectroscopy

Paraffin-embedded tissue serial sections (5 μm) were mounted on glass slides, deparaffinized using xylene followed by graded ethanol, and air-dried. No chemical stains were applied before O-PTIR analysis. O-PTIR measurements were performed in copropagation mode. The visible probe and IR pump powers at the sample were set to 52 and 2.5%, respectively, to minimize photodamage while maintaining adequate signal-to-noise. The probe focus was aligned to the tissue surface and maintained using the autofocus/feedback system when available. Point spectra were acquired from regions of interest identified by optical contrast and/or prior chemical maps. Spectra were collected over the fingerprint region (1800 to 1300 cm^−1^) at a spectral resolution of 2 cm^−1^. For chemical imaging, samples were scanned in the *XY* plane with a step size of 1 μm. PE detection focused on the -CH2- bending band near 1470 cm^−1^, with the amide I band (~1656 cm^−1^) used as a tissue reference. Ratio images (1470 cm^−1^/1656 cm^−1^) were generated to highlight PE-enriched regions relative to tissue background, and identical color scales were applied across comparable samples. Spectra were baseline-corrected using a rubber-band algorithm and vector-normalized over 1800 to 1300 cm^−1^. Spectra with poor signal-to-noise or obvious artifacts (e.g., tissue voids) were excluded on the basis of predefined criteria. Putative PE-positive regions were defined by elevated 1470/1656 ratios and confirmed by comparison with a PE reference spectrum acquired under identical experimental conditions. Identification was based on characteristic -CH2- deformation features at ~1470 to 1460 cm^−1^. Measurements were performed on two independent tissue sections, with four to seven regions analyzed per section. To minimize contamination, samples were handled using nonplastic tools where feasible and kept covered during transport and analysis.

### ChIP-seq data processing and ChIP-qPCR validation

Previous raw sequence reads were uniformly processed from SRX5142528 and SRX5028180 (*58*). Briefly, sequence read archive (SRA) files were converted to fastq format using the SRA Toolkit and then aligned to the appropriate reference genome with Bowtie2 using the mouse reference, mm10. Duplicate reads were removed using SAMtools. The resulting peaks were saved in a bigwig format. For visualization, the Peaks were visualized using the Integrative Genomics Viewer. On the basis of the identified peaks, selected DNA sequences targeting the central regions of these peaks were used for ChIP-qPCR to validate PPARα binding to Anxa2 enhancer and promoter regions. Chromatin was immunoprecipitated using anti-PPARα antibody (#PA5-85125, Invitrogen), followed by qPCR amplification of the regions associated with the peaks. Primer sequences are provided in table S3.

### Real-time qPCR analysis

Total RNA from liver tissues and hepatocytes were extracted using TRIzol (#15596018, Thermo Fisher Scientific, Waltham, MA). cDNA was synthesized using the iScript cDNA Synthesis Kit (#1708891, Bio-Rad, Hercules, CA). RT-qPCR was performed with gene-specific, exon-spanning primers (Integrated DNA Technologies, Coralville, IA) and PowerUp SYBR Green Master Mix (#A25742, Thermo Fisher Scientific) using the StepOnePlus real time PCR system (Thermo Fisher Scientific). Gene expression levels of 18*S* ribosomal RNA and/or *Rplp0* as a housekeeping control and *Anxa2* and *Acox1* were quantified in four to five samples. Relative expression was calculated using the ΔΔCt method, normalizing target gene expression to geometric mean of 18*S* and/or *Rplp0*. Primer sequences are listed in table S4.

### Public dataset analysis for PPARα agonism

Raw count data from GSE245069 (mouse treated with PS) and GSE282998 (mouse strain B6129SF2/J, treated with the PPARα agonist GW7647 including 0, 5, 10, and 20 mg) were imported into R and analyzed using the DESeq2 package (*59*). Genes with fewer than 10 total counts across all samples were excluded. Counts were normalized using the median-of-ratios method implemented in DESeq2. Normalized counts were extracted for downstream visualization and specific genes of interest, *Anxa2*. Publicly available microarray data from GSE8295 were also downloaded using the GEOquery package and processed with limma (*60*). The study used WT and *Ppara*-null 129S1/SvImJ mice, which were treated with either vehicle or WY14643 administered as a 0.1% CD supplement. Expression values were normalized by log_2_ transformed. Probe annotations were updated using Ensembl with the biomaRt package. A linear model was then fit to the expression data using the lmFit function of the limma package, and contrasts were defined to compare groups of interest using the probe, 1419091_a_at.

### Statistical analysis

Data are represented as means ± SD, unless otherwise indicated. For multiple groups, ordinary one-way ANOVA models with the Tukey post hoc procedure were used using GraphPad Prism software (Dotmatics, Boston, MA). For RT-qPCR, experimental and technical replicates were included for all samples to ensure data reliability and reproducibility. Further details on the study design, number of biological replicates, and statistical tests used are provided in the figure legends. ChatGPT (version 4o and 5, OpenAI) and Gemini (version 3 Flash, Google) were used as language tools to refine our writing, enhancing the clarity of our draft.
