# Supplementary material for: Spatial transcriptome mapping identifies Ppara-Anxa2 cross-talk in microplastic-induced hepatotoxicity
Source: Sci Adv. 2026 Jun 17;12(25):eaec8681. doi: 10.1126/sciadv.aec8681 (PMC13274612; doi:10.1126/sciadv.aec8681)
Supplement: Supplementary file 1 — Figs. S1 to S6 Legends for tables S1 to S18 [file sciadv.aec8681_sm.pdf]

Science Advances
AAAS

# Supplementary Materials for

## Spatial transcriptome mapping identifies *Ppara-Anxa2* cross-talk in microplastic-induced hepatotoxicity

Woncheol Jung *et al.*

Corresponding author: Aditya D. Joshi, aditya-joshi@tamu.edu; Tae Gyu Oh, taegyu-oh@ou.edu

*Sci. Adv.* **12**, eaec8681 (2026)
DOI: 10.1126/sciadv.aec8681

**The PDF file includes:**

Figs. S1 to S6
Legends for tables S1 to S18

**Other Supplementary Material for this manuscript includes the following:**

Tables S1 to S18

**Fig. S1**

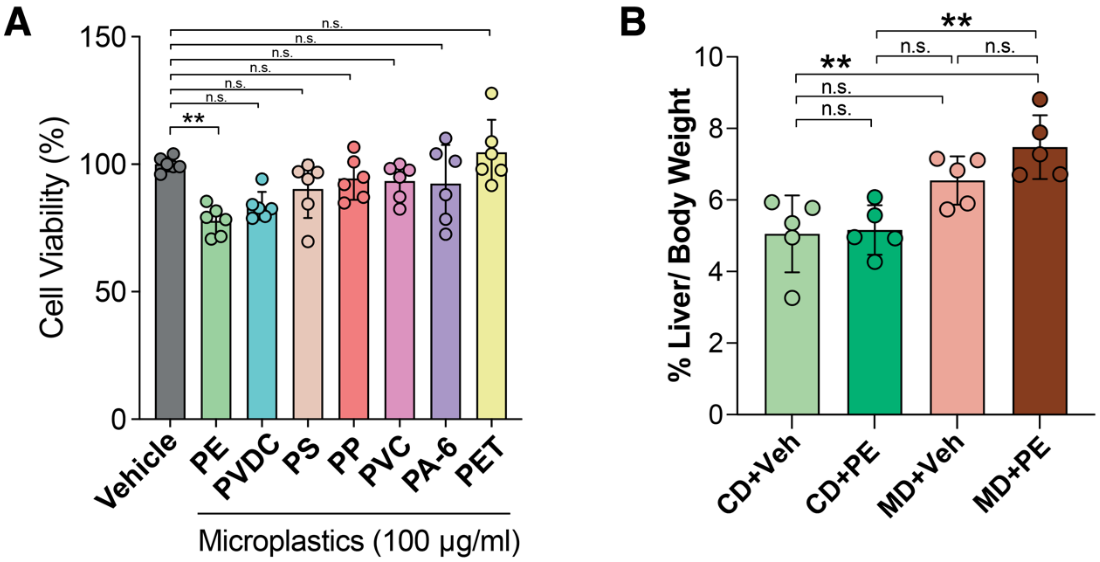


**Fig. S1: Polyethylene microplastics reduce liver cell viability without affecting the liver to body weight ratio, which is driven exclusively by the high-fat diet.** (**A**), *In vitro* viability of primary mouse hepatocytes following 24 hours exposure to various microplastic types, including Polyethylene (PE), Polyvinylidene chloride (PVDC), Polystyrene (PS), Polypropylene (PP), Polyvinyl chloride (PVC), Polyamide-6 (PA-6), and Polyethylene terephthalate (PET). Cells were treated with 100 µg/ml of MPs. A detailed description of the experimental setup and reagent sources is provided in the Methods section. (**B**), Liver-to-body weight ratio (%) across treatment groups (n = 5 per group). Data are presented as mean ± SD. Statistical significance was determined using ordinary one-way ANOVA with Tukey's multiple comparisons test. ns, not significant; *, $P < 0.05$; **, $P < 0.01$; ***, $P < 0.001$.

Fig. S2

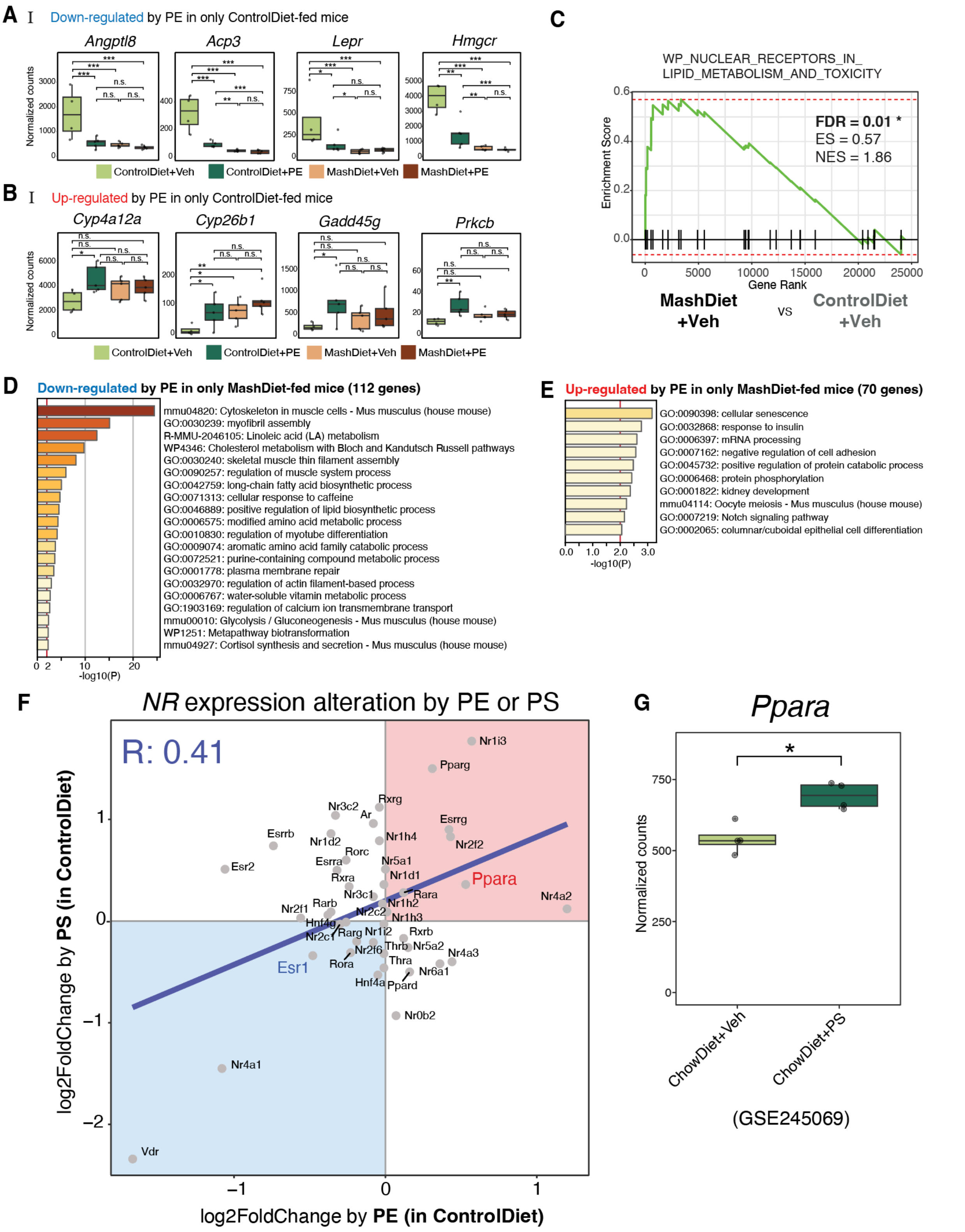


**Fig. S2: High-fat diet and PE microplastics drive divergent gene expression profiles with cross-plastic validation of *Ppara* as a key responsive nuclear receptor.** RNA sequencing of liver tissues from mice

treated with PE microplastics under control or MASH diets reveals transcriptomic changes linked to disrupted lipid metabolism and activation of hepatic toxicity pathways. (**A-B**), CD-specific DEGs that are either downregulated (A) or upregulated (B) in responsive to PE excluding genes also affected under MD conditions. Boxplots (ggplot2) represent median, interquartile range (IQR), and whiskers (1.5x IQR). (**C**), GSEA of the nuclear receptor-regulated lipid metabolism and toxicity gene set comparing MD+Veh versus CD+Veh, highlighting transcriptional pathways altered by MD treatment. (**D-E**), Using Enrichr, Gene Ontology (GO) enrichment analysis of the 182 differentially expressed genes from Figure 2C, which were separated into downregulated (D) and upregulated (E) sets. (**F**), Fold change in nuclear receptor gene expression in response to PS or PE treatment, in CD condition. In order to compare PS and PE effect, GSE245069 fastq files including PS treated mouse liver were downloaded processed with the same pipeline. (**G**), PS exposure significantly increased *Ppara* expression in CD-fed mice. Data were obtained from GSE245069, and the RNA-seq dataset was processed using the same analytical pipeline as applied in our study. Statistics and abbreviations: Data are presented as the means ± SD. For all comparative analyses, significance was determined using DESeq2-derived FDR-adjusted *P* values. ns, not significant; *, FDR <0.05; **, FDR <0.01; ***, FDR <0.001. GSEA, Gene Set Enrichment Analysis; FDR, false discovery rate; ES, enrichment score; NES, normalized enrichment score; CD, control diet; MD, MASH diet; Veh, vehicle; PE, polyethylene; PS, polystyrene.

Fig. S3

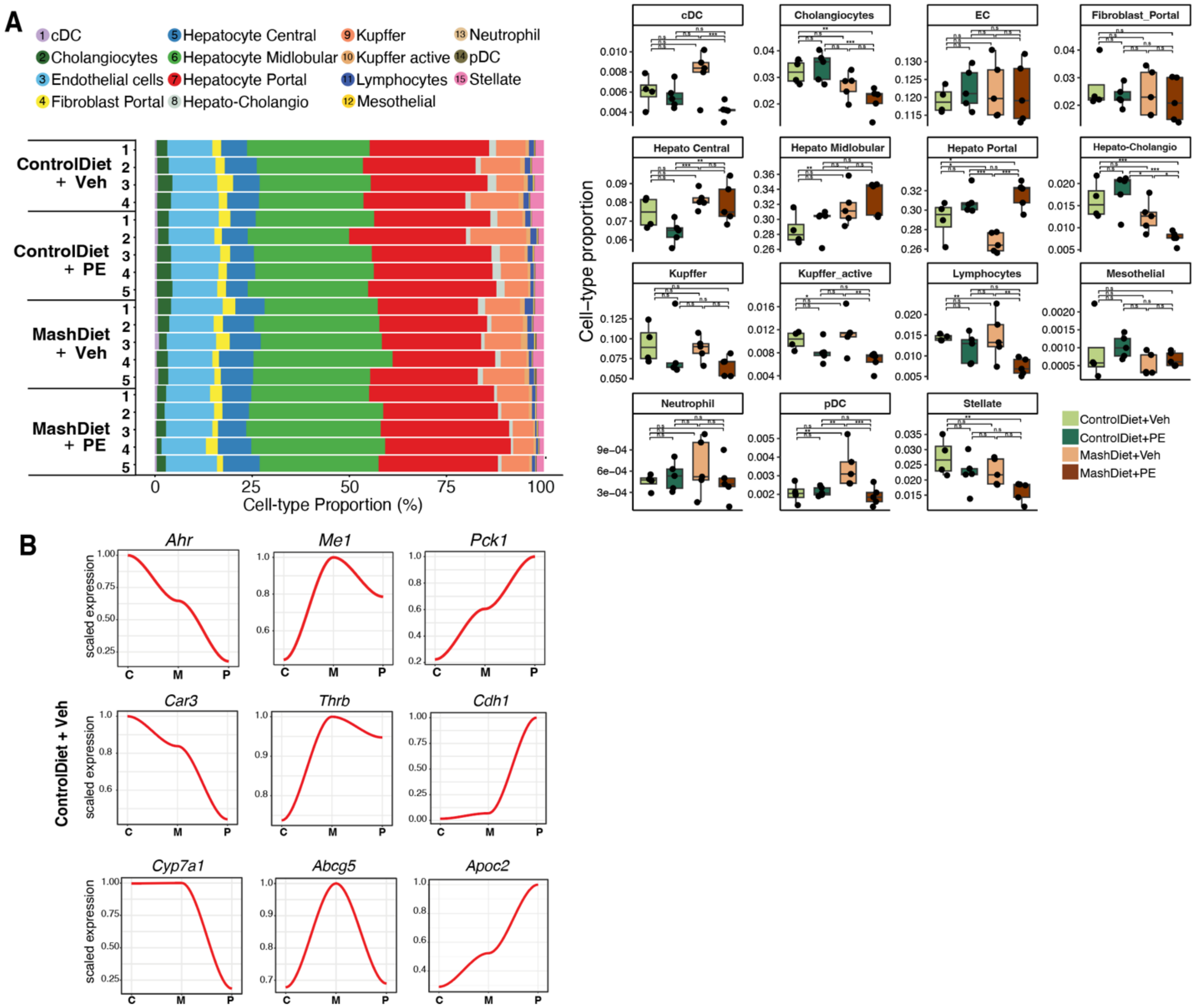


**Fig. S3**: **Characterization of hepatic cell-type proportions and zonal expression profiling of hepatocyte markers defined by spatial transcriptomics.** Spatial transcriptomic profiling of liver sections from mice fed either a microplastic-enriched CD or MD revealed distinct patterns of liver zonation and NR gene expression. (**A**), Proportions of identified cell types across experimental samples, including CD+Veh, CD+PE, MD+Veh, and MD+PE. Each bar represents the average cell type proportion derived from each sample. The same color code for cell types as in Figure 3C was applied. Cell type labels include abbreviations as follows: cDC, conventional dendritic cells; Hepato-Cholangio, intermediate cells with features of both hepatocytes and cholangiocytes; pDC, plasmacytoid dendritic cells. Changes in cell-type

proportions across experimental conditions. Proportional data, bounded between 0 and 1, were modeled using beta regression. Pairwise comparisons between conditions were performed using estimated marginal means (EMMs) derived from the fitted models, with *P* values adjusted for multiple testing using the Holm method. Individual points represent biological replicates (samples). Boxplots indicate the median, interquartile range (IQR), and whiskers extending to 1.5 time the IQR. (**B**), Expression patterns of hepatocyte subtype marker genes across liver zones in the control group. Zonal hepatocyte identity was validated in central (C) with *Ahr*, *Car3,* and *Cyp7a1* expression, midlobular (M) with *Me1*, *Thrb*, and *Abcg5* expression, and portal (P) with *Pck1*, *Cdh1*, and *Apoc2* expression. Statistics and abbreviations: Data are presented as means ± SD. ns, not significant; *, $P < 0.05$; **, $P < 0.01$; ***, $P < 0.001$. CV, central vein; PV, portal vein. CD, control diet; MD, MASH diet; Veh, vehicle; PE, polyethylene.

**Fig. S4**

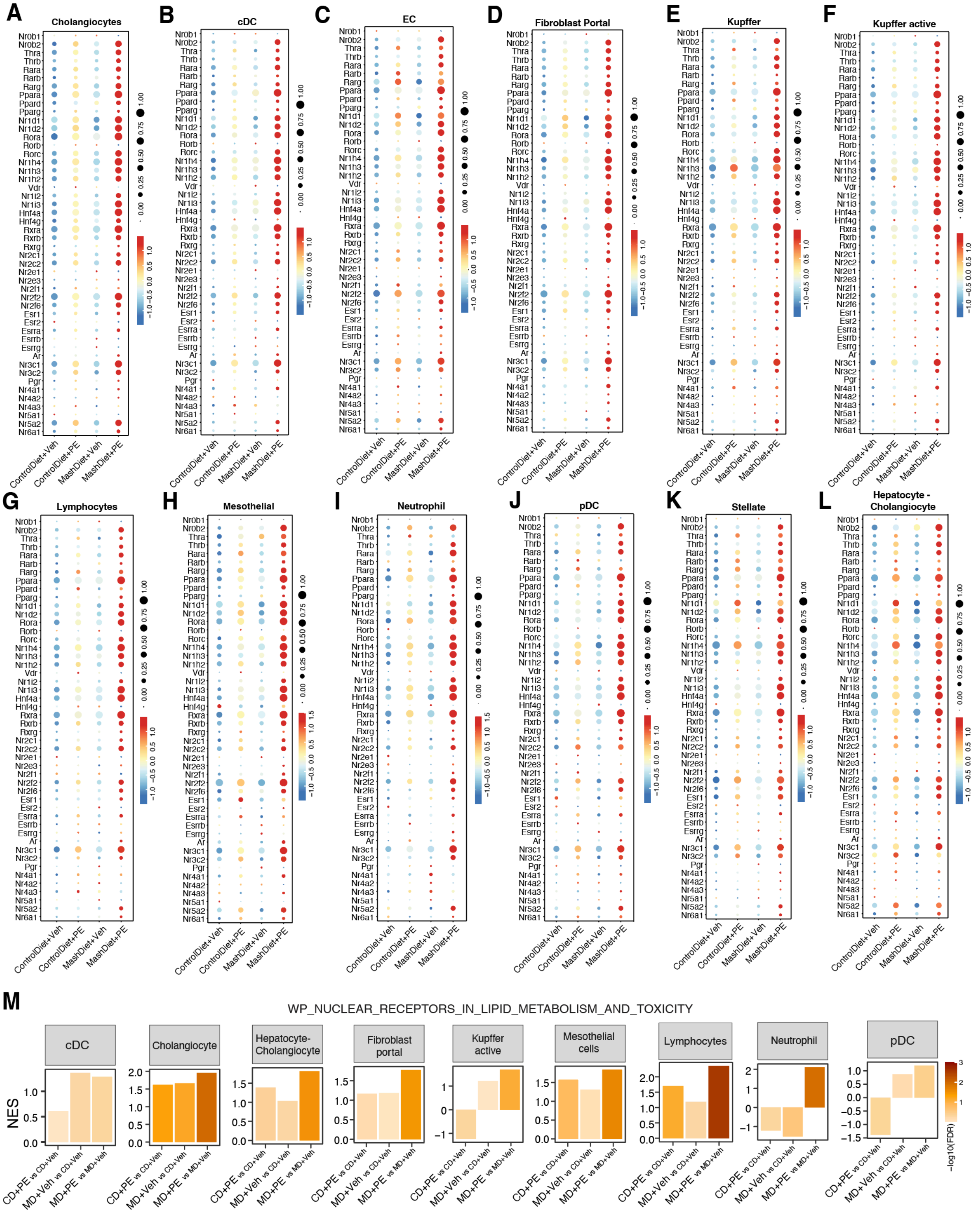


**Fig. S4: Spatial transcriptome-derived single cell data reveals differential gene expression and metabolic pathway alterations in distinct liver cell populations under varying dietary conditions. (A-**

**L**), Comparison of NR gene expression across treatment groups in various cell types. Color indicates relative gene expression levels, while dot size reflects the proportion of cells expressing each gene. Cell type labels include abbreviations as follows: cDC, conventional dendritic cells; pDC, plasmacytoid dendritic cells. (**M**), GSEA of the NR lipid metabolism and toxicity gene set across various liver cell types for three comparisons: (i) CD+PE vs. CD+Veh; (ii) MD+Veh vs. CD+Veh; and (iii) MD+PE vs. MD+Veh. Y-axis indicates normalized enrichment levels, while color reflects the significance in each cell type. Abbreviation: GSEA, Gene Set Enrichment Analysis; DEG: differentially expressed gene, FDR: false discovery rate, ES: enrichment score, NES: normalized enrichment score; CD, control diet; MD, MASH diet; Veh, vehicle; PE, polyethylene.

**Fig. S5**

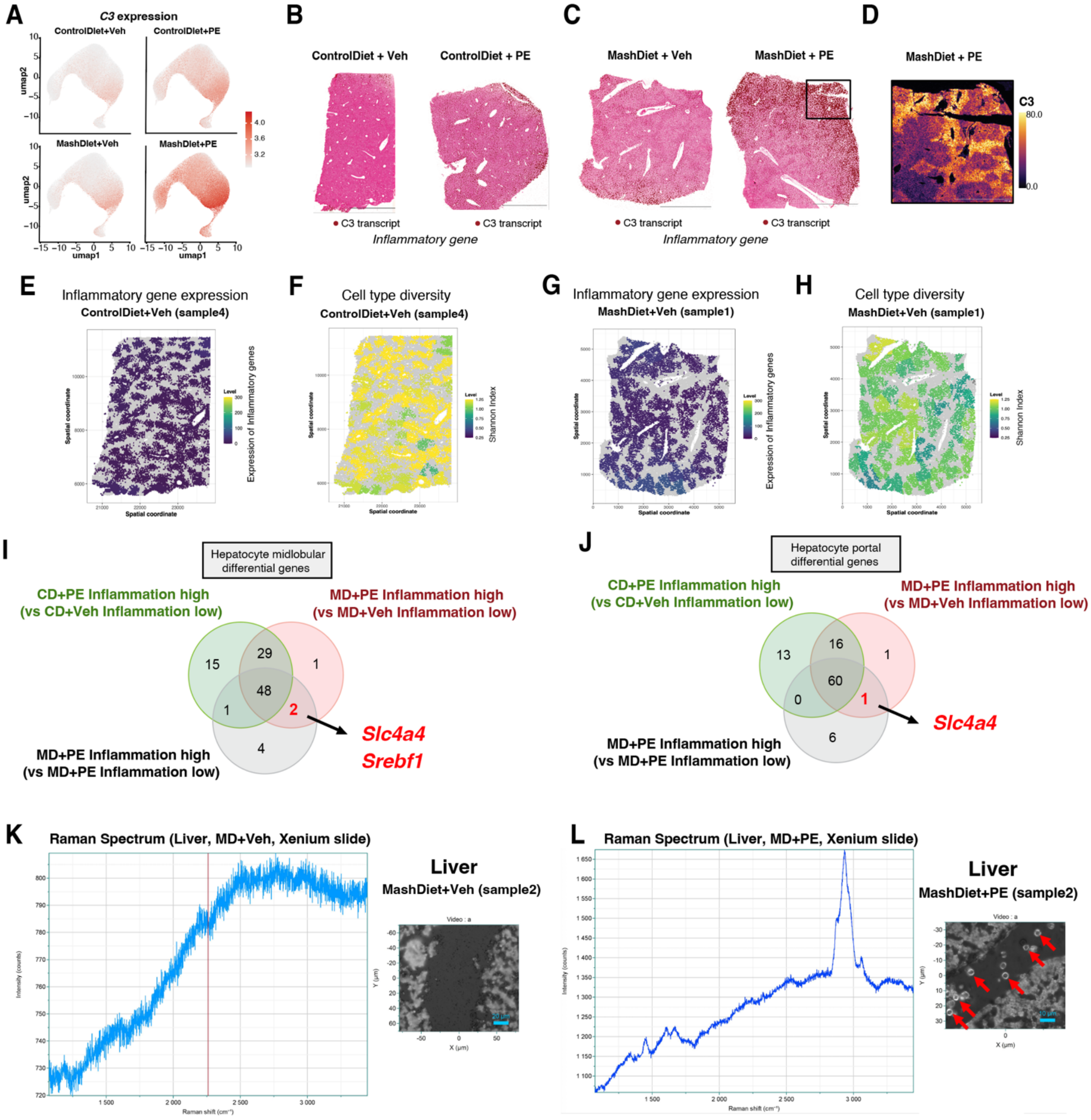


**Fig. S5: Spatial transcriptome analysis revealed regions of inflammation and tissue damage in mice treated with or without microplastics, uncovering altered cell type diversity and changes in gene expression patterns.** (**A**), UMAP visualization of spatial transcriptome-derived single cells, with color intensity indicating Complement Component 3 (*C3*) expression levels, a marker of inflammatory response, across distinct hepatocyte subtypes and cholangiocytes from all experimental conditions. (**B-C**),

Visualization of C3 transcripts on top of histological image in (B) CD with or without PE and (C) MD with or without PE conditions. (**D**), Density plot (20 µm bin size) of *C3* transcripts within a zoomed region of the MD+PE sample. (**E-H**), Identification of Inflammatory regions based on a multi-gene set (*Alox5ap*, *C3*, *Dnase1l3*, *F3*, *Hp*, *Nupr1*, *Reg3g*, *Ccr7*, and *Il6*) and cell-type diversity (Shannon index) within spatially clustered central and midlobular areas of CD+Veh and MD+Veh samples. (**I-J**), Venn diagram illustrating the overlap of DEGs among three inflammation-focused comparisons in midlobular hepatocyte cells (I) or portal hepatocytes (J). DEGs were identified using the *FindMarkers* function ($|\log_2 FC| \geq 0.58$, FDR < 0.05, min. expressing cell proportion ≥ 0.25). (**K-L**), Raman spectroscopic analysis of liver tissue on Xenium slide. Representative spectra and optical micrographs show the absence of PE-associated Raman peaks in MD+Veh control (K) and the presence of prominent C-H stretching peaks characteristic of PE particles in MD+PE group(L). The corresponding optical image shows spherical particulate structures (red arrows) within the scanned region where spectra were collected. Scale bar: 20 µm (K), 10 µm (L). Abbreviation: CD, control diet; MD, MASH diet; Veh, vehicle; PE, polyethylene.

Fig. S6

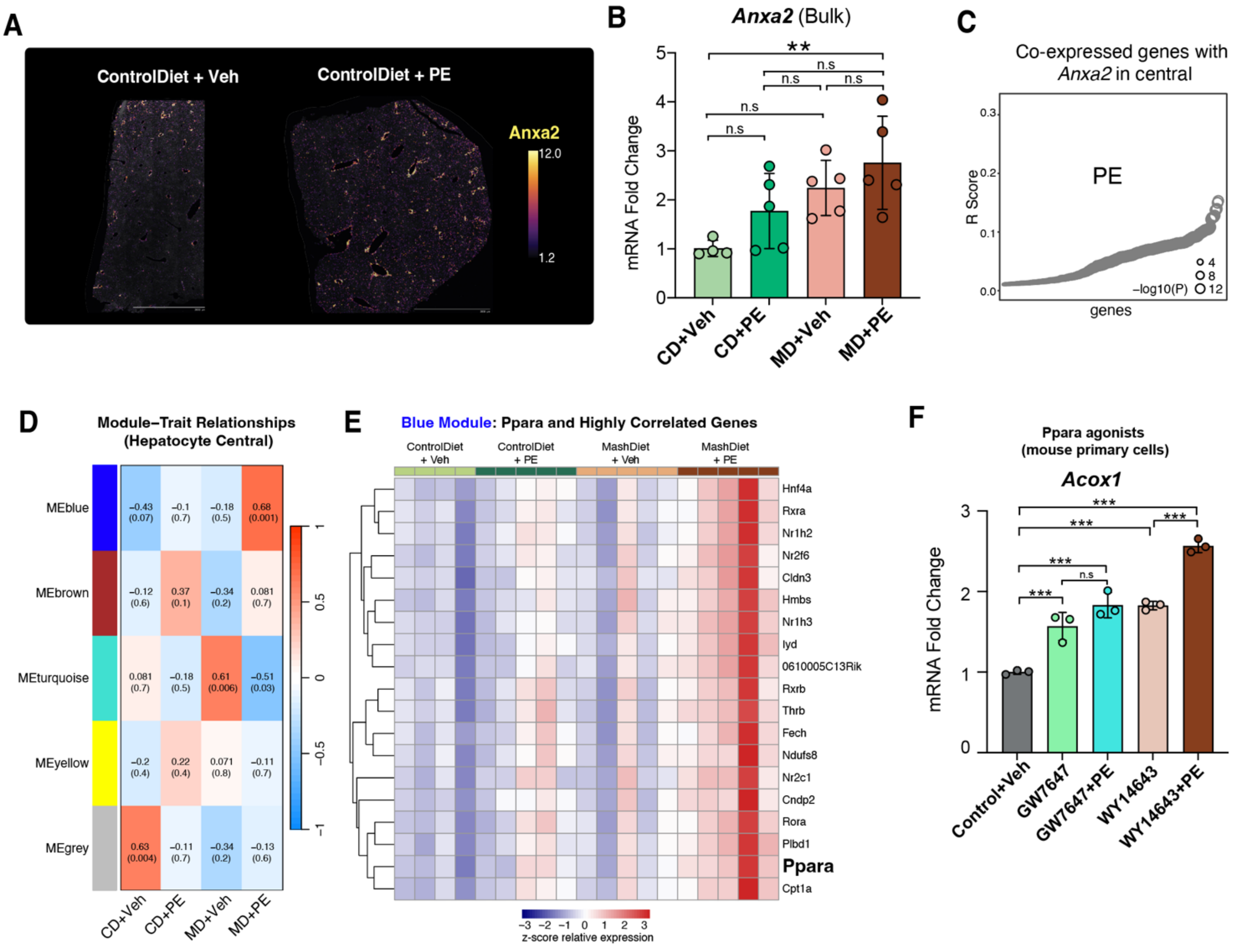


**Fig. S6: *Ppara* is correlated with *Anxa2* expression in the liver in response to microplastic and dietary interventions.** (**A**), Xenium-derived density plots of of *Anxa2* transcripts in CD+Veh and CD+PE samples (bin size: 20 µm). (**B**), RT-qPCR quantification of *Anxa2* expression in response to PE and diet-induced liver injury, normalized to the geometric mean of *Rplp0* and *18S*. (**C**), Spearman correlation analysis of *Anxa2* in central hepatocytes (CD+PE samples, relative to the CD+Veh group); circle size denotes -$\log_{10}P$. (**D**), Weighted Gene Co-expression Network Analysis (WGCNA) outcome showing a module-trait relationship heatmap with the correlation between identified gene co-expression modules (y-axis) and experimental conditions (x-axis). Each cell contains the Pearson correlation coefficient and the corresponding *P* value, with the color scale representing the strength of the correlation (red indicating

positive correlation; blue indicating negative). The Blue module exhibits a significant positive correlation specifically with the MD+PE condition (r =0.68, p = 0.001), indicating a distinct transcriptional program associated with this phenotype. (**E**), Expression profile of this Blue module (MEblue) across experimental groups, where the y-axis represents the Module Eigengene (ME) value, a summary measure of the gene expression profile for the module. The Blue module is significantly upregulated in the MD+PE group compared to Controls and CD+PE. Key driver genes within this module, including *Ppara*, are highlighted, confirming their central role in the gene regulatory network underlying the MD+PE phenotype. (**F**), Induction of *Acox1* (PPARα target gene) by PPARα agonists (GW7647 and WY14643, 10 μM) in primary mouse cells after 24 hours. Statistics and abbreviations: Data are presented as mean ± SD. Significance was determined using ordinary one-way ANOVA with Tukey's multiple comparisons test or Spearman correlation analysis. ns, not significant *, $P < 0.05$; **, $P < 0.01$; ***, $P < 0.001$. CD, control diet; MD, MASH diet; Veh, vehicle; PE, polyethylene.

Supplementary table captions

Tables S1 to S18

| | |
|---|---|
| Table S1 | Mouse liver toxicity |
| Table S2 | Multi mouse 100 add-on genes |
| Table S3 | ChIP-qPCR Primers and Antibody |
| Table S4 | RT-qPCR primers |
| Table S5 | BULK_RNA-seq_G1vsG2, G1vsG3, G1vsG4 (pairwise) |
| Table S6 | BULK_RNA-seq_G2vsG3, G2vsG4, G3vsG4 (pairwise) |
| Table S7 | BULK_RNA-seq_Interaction_Analysis |
| Table S8 | BULK_Nuclear_Receptor_Expression (Sample-Based) with the heatmap |
| Table S9 | Spatial single-cell CD cell type marker genes |
| Table S10 | Spatial single-cell_G1vsG2, G1vsG3, G1vsG4 (pairwise, Sample-Based and Cell-Based) |
| Table S11 | Spatial single-cell_G2vsG3, G2vsG4, G3vsG4 (pairwise, Sample-Based and Cell-Based) |
| Table S12 | Spatial single-cell_Interaction_Analysis |
| Table S13 | Diversity and Proportion Statistics (Central/Midlobular region) (Sample-Based and Cluster-Based Regression Analysis) |
| Table S14 | Spatial DEG Kupffer (Central/Midlobular region) |
| Table S15 | Spatial DEG Hepato_Central (Central/Midlobular region) |
| Table S16 | Spatial DEG Hepato_Midlobular (Central/Midlobular region) |
| Table S17 | Spatial DEG Hepato_Portal (Portal region) |
| Table S18 | Ppara ChIP-qPCR & Anxa2 RT-qPCR |
